# Severity of Systemic Inflammatory Response Syndrome Affects the Blood Levels of Circulating Inflammatory-Relevant MicroRNAs

**DOI:** 10.3389/fimmu.2017.01977

**Published:** 2018-02-05

**Authors:** Stefano Caserta, Manuela Mengozzi, Florian Kern, Sarah F. Newbury, Pietro Ghezzi, Martin J. Llewelyn

**Affiliations:** ^1^Brighton and Sussex Medical School, University of Sussex, Falmer, United Kingdom; ^2^Brighton and Sussex University Hospitals NHS Trust, Brighton, United Kingdom

**Keywords:** systemic inflammatory response syndrome, sepsis, microRNA, inflammation, immune cells, sequential organ failure assessment score, miR-378, miR-30

## Abstract

The systemic inflammatory response syndrome (SIRS) is a potentially lethal response triggered by diverse forms of tissue injury and infection. When systemic inflammation is triggered by infection, the term sepsis is used. Understanding how inflammation is mediated and regulated is of enormous medical importance. We previously demonstrated that circulating inflammatory-relevant microRNAs (CIR-miRNAs) are candidate biomarkers for differentiating sepsis from SIRS. Here, we set out to determine how CIR-miRNA levels reflect SIRS severity and whether they derive from activated immune cells. Clinical disease severity scores and markers of red blood cell (RBC) damage or immune cell activation were correlated with CIR-miRNA levels in patients with SIRS and sepsis. The release of CIR-miRNAs modulated during SIRS was assessed in immune cell cultures. We show that severity of non-infective SIRS, but not sepsis is reflected in the levels of miR-378a-3p, miR-30a-5p, miR-30d-5p, and miR-192-5p. These CIR-miRNA levels positively correlate with levels of the redox biomarker, peroxiredoxin-1 (Prdx-1), which has previously been shown to be released by immune cells during inflammation. Furthermore, *in vitro* activated immune cells produce SIRS-associated miR-378a-3p, miR-30a-5p, miR-30d-5p, and miR-192-5p. Our study furthers the understanding of the origin, role, and trafficking of CIR-miRNAs as potential regulators of inflammation.

## Introduction

The systemic inflammatory response syndrome [SIRS (1)] can be triggered by diverse forms of injury including burns, ischemia, autoimmune diseases, injuries including surgery and infection. The severity of illness seen in SIRS varies widely but severe SIRS accompanied by multiple organ dysfunction syndrome (MODS), especially in the setting of sepsis (a condition which shares common clinical manifestations with SIRS), may be lethal (2). Even with optimal medical care, mortality rates in severe sepsis increase to around 50% (3). In this respect, clinical scores, such as the sequential organ failure assessment [SOFA (4)] and the acute physiology and chronic health evaluation II [APACHE II (5)], are useful to evaluate SIRS severity and patient mortality risk.

During SIRS, immune cell activation spreads to the whole body driving severe immunopathology (1, 6, 7). Damage- and/or pathogen-associated molecular pattern molecules (respectively, DAMPs and PAMPs) released after injury (or infection) initiate, *via* toll-like receptor (TLR) signals (6, 8), an activation cascade in immune and endothelial cells leading to inflammatory cytokine production [e.g., tumor necrosis factor (TNF) α, IL-1, IL-6, and IL-8], in a so-called “cytokine storm” (9, 10). Activated inflammatory cells migrate to affected organs and release secondary inflammatory mediators (1), including reactive oxygen species (ROS) and nitrogen species (11–15), resulting in oxidative stress (16, 17). Inflammatory cell migration to tissues and endothelial cell activation (18) lead to disseminated intravascular coagulation (19) aggravating organ damage. Increased levels of TNFα, IL-1, and IL-6 have been reported to occur more often in sepsis than SIRS (1, 20–22). Anti-inflammatory mediators (e.g., TGFβ and cytokine antagonists) are also present in plasma during SIRS, with soluble cytokine receptors often found at concentration higher than the respective cytokines (20, 23), hence compensatory anti-inflammatory responses [CAR (24)] may be activated during SIRS. Understanding the responses which occur in non-infective inflammation and sepsis and how they are regulated will aid development of diagnostics and therapeutics in these major inflammatory diseases.

MicroRNAs (miRNAs) are small (~23 nt) RNAs that function as post-transcriptional gene regulators (25, 26). By annealing to complementary sequences in the 3′ untranslated region (3′ UTR) of target mRNAs, they reduce expression of specific proteins by promoting target degradation or inhibition of translation (26). The role of miRNAs in SIRS remains unclear, although many inflammatory cytokines, mediators, and their regulators are miRNA targets (27). Approximately 100–200 miRNAs are found in human blood in health and disease (28–30). Previous studies (31, 32) suggest that exogenous miRNAs may be detected by TLRs (33, 34), particularly TLR7 (32), as potential DAMPs leading to inflammatory signals. We previously identified a pool of circulating inflammatory-relevant miRNA (CIR-miRNAs) that robustly distinguish sepsis from non-infective SIRS (35). We were the first to find that, in these conditions, CIR-miRNA levels inversely correlate with those of inflammatory cytokines (35), suggesting that they may be part of the CAR.

To extend these observations, we set out to determine how CIR-miRNAs reflect SIRS severity and whether they are likely to arise directly from immune activation. In this study, we investigated the correlation of plasma levels of CIR-miRNAs with disease severity and other inflammatory parameters. We found that SIRS severity significantly impacts on the levels of miR-378a-3p, miR-30a-5p, miR-30d-5p, and miR-192-5p. These CIR-miRNAs are unlikely to derive from red blood cell (RBC) damage consequent to hemolysis or coagulopathy. Instead their levels positively correlate with levels of the redox biomarker, Prdx-1, which is released by immune cells during inflammation (36, 37). Finally, we show that activated immune cells produce SIRS-associated miR-378a-3p, miR-30a-5p, miR-30d-5p, and miR-192-5p, *in vitro*. Our study has implications for furthering the understanding of the origin and role and trafficking of CIR-miRNAs in SIRS, sepsis, and potentially other inflammatory disease.

## Materials and Methods

### Patients and Healthy Donors

The patient population used in this study was previously described in detail (35, 38). Briefly, patients comprised unselected adult admissions to a mixed medical/surgical intensive/high-dependency care unit (ICU/HDU) at an English acute hospital (Brighton and Sussex University Hospitals NHS Trust). Patients were categorized as having non-infective SIRS (*n* = 44) or sepsis (*n* = 29), following standard criteria (38). SIRS severity was defined as: severe (SOFA ≥ 6) and non-severe (SOFA ≤ 3); patients with intermediate SOFA scores of 4–5 were excluded. Only patients with abdominal sepsis were included in this study. Blood samples were collected within <6 h from admission and time of sample collection did not affect levels of CIR-miRNAs (35). Healthy donors were recruited at Brighton and Sussex Medical School (BSMS, University of Sussex, Falmer, Brighton, UK). Refer to Materials and Methods in Supplementary Material for further details, including details of any medication targeting inflammation that patients were taking at the time of admission.

### Hemoglobin, Prdx-1, and Cytokine Analysis in Plasma Samples

Red blood cell lysis can bias miRNA content in plasma (39, 40). The concentration of free hemoglobin (Hb) was independently measured in patient plasma by the Harboe spectrophotometric method (41, 42) and hemolytic samples (43–45) were excluded, as carried out previously (35). Prdx-1 plasma levels were independently measured using an ELISA kit for human Prdx-1 (#ABIN418422) from Antibodies Online (Aachen, Germany). Plasma cytokines (IL-6, IL-8) and anti-/inflammatory mediators [pro-calcitonin (PCT), C-reactive protein (CRP), and soluble CD25 (sCD25)] were measured on Luminex LX200 and ELISA (sCD25) as previously described (38).

### Human Samples and Primary Cell Cultures

Human peripheral blood mononuclear cells (PBMCs) from healthy donors were freshly isolated by centrifugation over Ficoll–Hypaque density gradient as previously described (46). Cells were washed in sterile PBS (ThermoFisherScientific) and counted with 0.1% Trypan Blue cell viability exclusion dye (Sigma). Thereafter, 20 × 10^6^ viable PBMCs were resuspended in 10 ml (2 × 10^6^ cells/ml) of complete media: RPMI containing 100 IU/ml penicillin, 100 µg/ml streptomycin, 2 mM l-glutamine (all from ThermoFisherScientific) and 10% of exosome-depleted, heat-deactivated fetal calf serum (System Biosciences). PBMCs were stimulated with the bacterial superantigen (SAg), streptococcal pyrogenic exotoxin K/L [SPE-K/L (47)] as described before (46), in parallel to unstimulated control cultures (24-well plates, 1 ml/well). After 5 days, cells were harvested and assayed for viability with Trypan-blue (0.1%) (Figure S7 in Supplementary Material). Equal volumes of culture supernatants were then recovered, frozen at −80°C and total RNA was then extracted as described below.

### RNA Extraction and MicroRNA Real-Time qPCR Array

Plasma and supernatant RNA was extracted using the miRCURY™ RNA isolation—biofluids kit (Exiqon, Denmark). After thawing on ice, an RNA spike-in template mixture was added to the samples. Eight milliliters of supernatant per sample was mixed with 2.4 ml of Lysis solution BF containing 16.67 µg/ml of MS2 bacteriophage RNA (UniSp6), prior to RNA purification, as specified by the manufacturer’s instructions. Extracted total RNA was stored in a −80°C freezer. RNA was reverse transcribed (RT) and cDNA analyzed using the miRCURY LNA™ Universal RT miRNA PCR, Polyadenylation, and cDNA synthesis method. For plasma samples derived from patients (≥8 biological replicates), each microRNA was assayed by qPCR (miRNA Ready-to-Use PCR, Pick-&-Mix using ExiLENT SYBR^®^ Green master mix) in two independent technical repeats, including negative controls (no-template from the RT reaction) using a LightCycler^®^ 480 Real-Time PCR System (Roche). SAg stimulation experiments were performed as 10 independent biological replicates. Assays returning three crossing point (Cp) values less than the negative control and Cp < 37 were accepted. The stability values of candidate normalizers were assessed using the “NormFinder” software (48). Any qPCR data were normalized to the average Cp of internal normalizers [miR-320a and miR-486-5p (35)] in plasma samples and, in the case of culture supernatants, the Cp of normalizer spike-in (UniSp6); (delta Cp, dCp = normalizer Cp − assay Cp). Refer to the Materials and Methods in the Supplementary Material for further details.

### Statistical Analyses

Datasets were analyzed using the GraphPad Prism 6 and/or IBM SPSS Statistics 22 software. The D’Agostino and Pearson omnibus and Shapiro–Wilk tests were used to test normal data distribution; data were considered normally distributed only if they passed both tests. If not normally distributed, medians with interquartile ranges (IQRs, rather than means and SD) are shown and Mann–Whitney *U* Test (rather than *t*-tests) was used to calculate *p* values in 2-group comparisons. Correlations between SOFA and APACHE II scores and plasma levels of CIR-miRNAs and inflammatory cytokines/mediators were evaluated using the Spearman rho (ρ) and significances of the correlations determined as indicated. Generally, 0.35 ≤ ρ ≤ 1 or −1 ≤ ρ ≤ −0.35 were considered as moderate-to-strong correlation trends (49, 50). The confidence in the predictive value of each correlation was assessed by individual *p* values; in general, correlations trends were considered significant only if *p* ≤ 0.05 (51). In addition, for the qPCR miRNA array dataset, a Benjamini–Hochberg multiple comparison correction (52) was used to control for the number of false positives, using false discovery rates of 15% (Tables 1 and 2; Tables S1 and S2 in Supplementary Material) and 5% (Tables 3 and 4) which correspond to a ~1/6 and 1/20 chance of false positives, respectively. Such correction excludes that the significance of correlation of any parameter (such as disease severity, free Hb and Prdx-1) with any of the 43 miRNA tests run in parallel was not simply due to the chance of multiple testing. Unless stated differently in figure legends, levels of significance were assigned as: *p ≤ 0.05; **p ≤ 0.005; and ***p ≤ 0.0005. MiRNA and cytokine, Hb, and Prdx-1 analyses were conducted blind to the clinical data.

**Table 1:** Correlations of circulating inflammatory-relevant microRNAs (CIR-miRNAs) with severity of systemic inflammatory response syndrome (SIRS) as detected by sequential organ failure assessment (SOFA).

MicroRNA species^a^	SOFA Spearman correlation (ρ)^b^	Correlation significance *p*^c^	Benjamini–Hochberg (BH) rank	BH critical value (FDR 15%)
**miR-378a-3p**	0.491	0.00084	1*	0.00349
**miR-30a-5p**	0.433	0.00370	2*	0.00698
**miR-30d-5p**	0.412	0.00609	3*	0.01047
**miR-192-5p**	0.378	0.01253	4*	0.01395
**miR-122-5p**	0.359	0.01799	5	0.01744
**miR-101-3p**	0.351	0.02092	6	0.02093
**miR-21-5p**	0.336	0.02769	7	0.02442
**miR-148a-3p**	0.309	0.04357	8	0.02791
miR-10b-5p	0.283	0.06601	9	0.03140
miR-532-5p	0.285	0.07054	10	0.03488
**miR-22-3p**	0.268	0.08248	11	0.03837
miR-143-3p	0.266	0.08468	12	0.04186
miR-23a-3p	0.255	0.09926	13	0.04535
miR-320a	0.251	0.10514	14	0.04884
miR-486-5p	−0.251	0.10514	15	0.05233

*The top 15 performing miRNAs are shown. Significant correlations with disease severity are highlighted in bold black (SIRS) or red (sepsis). Refer to Table S4 in Supplementary Material for the full dataset*.

*^a^Green-shadowed cells indicate miRNAs that passed the BH correction for multiple comparisons*.

*^b^Blue- and violet-shadowed cells indicate miRNAs that returned positive and negative correlations (ρ ≥ 0.2 or ρ ≤ −0.2), respectively*.

*^c^Brown- and red-shadowed cells indicate miRNAs that returned significant correlations (p ≤ 0.05) and additionally passed the correction for multiple comparisons, respectively*.

**Table 2:** Correlations of circulating inflammatory-relevant microRNAs (CIR-miRNAs) with severity of sepsis as detected by sequential organ failure assessment (SOFA).

MicroRNA species^a^	SOFA Spearman correlation (ρ)^b^	Correlation significance *p*^c^	Benjamini–Hochberg(BH) rank	BH critical value (FDR 15%)
**miR-22-3p**	0.447	0.01942	1	0.00349
**miR-191-5p**	−0.432	0.02436	2	0.00698
**miR-375**	0.590	0.02633	3	0.01047
miR-151a-3p	−0.354	0.06990	4	0.01395
miR-146a-5p	−0.333	0.08966	5	0.01744
miR-103a-3p	−0.299	0.12992	6	0.02093
**miR-378a-3p**	0.282	0.16305	7	0.02442
let7b-5p	−0.269	0.17521	8	0.02791
**miR-122-5p**	0.262	0.19539	9	0.03140
let7i-5p	−0.252	0.20556	10	0.03488
**miR-192-5p**	0.228	0.25220	11	0.03837
miR-23a-3p	−0.224	0.26149	12	0.04186
miR-92b-3p	−0.304	0.27048	13	0.04535
miR-10a-5p	−0.275	0.28483	14	0.04884
miR-28-3p	−0.192	0.34735	15	0.05233

*The top 15 performing miRNAs are shown. Significant correlations with disease severity are highlighted in bold black (SIRS) or red (sepsis). Refer to Table S5 in Supplementary Material for the full dataset*.

*^a^Green-shadowed cells indicate miRNAs that passed the BH correction for multiple comparisons*.

*^b^Blue- and violet-shadowed cells indicate miRNAs that returned positive and negative correlations (ρ ≥ 0.2 or ρ ≤ −0.2), respectively*.

*^c^Brown- and red-shadowed cells indicate miRNAs that returned significant correlations (p ≤ 0.05) and additionally passed the correction for multiple comparisons, respectively*.

**Table 3:** Correlations of circulating inflammatory-relevant microRNAs (CIR-miRNAs) with free hemoglobin levels in non-infective systemic inflammatory response syndrome (SIRS).

MicroRNAspecies^a^	Hb Spearman correlation (ρ)^b^	Correlation significance *p*^c^	Benjamini Hochberg(BH) rank	BH critical value (FDR 5%)
**miR-21-5p**	−0.6308	5.78E-06	1	0.00116
miR-10b-5p	−0.5297693	2.59E-04	2	0.00233
miR-320a	−0.5080983	0.000504483	3	0.00349
miR-486-5p	0.5080983	0.000504483	4	0.00465
**miR-375**	−0.5562014	0.000521672	5	0.00581
miR-451a	0.5024351	0.000596273	6	0.00698
miR-30e-3p	−0.4792892	0.001521716	7	0.00814
**miR-30a-5p**	−0.4631706	0.001761638	8	0.00930
miR-92b-3p	−0.4986164	0.001966918	9	0.01047
**miR-22-3p**	−0.4430098	0.002929346	10	0.01163
**miR-122-5p**	−0.4416506	0.00302822	11	0.01279
miR-28-3p	−0.4402159	0.003135754	12	0.01395
miR-146a-5p	−0.4328161	0.00374532	13	0.01512
**miR-192-5p**	−0.4219428	0.004828522	14	0.01628
**miR-101-3p**	−0.4151471	0.005636129	15	0.01744

*The top 15 performing miRNAs are shown. miRNAs significantly correlating with disease severity (as by sequential organ failure assessment) are highlighted in bold black (SIRS) or red (sepsis). Refer to Table S6 in Supplementary Material for the full dataset*.

*^a^Green-shadowed cells indicate miRNAs that passed the BH correction for multiple comparisons*.

*^b^Blue- and violet-shadowed cells indicate miRNAs that returned positive and negative correlations (ρ ≥ 0.2 or ρ ≤ −0.2), respectively*.

*^c^Brown- and red-shadowed cells indicate miRNAs that returned significant correlations (p ≤ 0.05) and additionally passed the correction for multiple comparisons, respectively*.

**Table 4:** Correlations of circulating inflammatory-relevant microRNAs (CIR-miRNAs) with peroxiredoxin-1 levels in non-infective systemic inflammatory response syndrome (SIRS).

MicroRNA species^a^	Prdx1 Spearman correlation (ρ)^b^	Correlation significance *p*^c^	Benjamini Hochberg(BH) rank	BH critical value (FDR 5%)
**miR-192-5p**	0.590909	3.02E-05	1	0.00116
**miR-30a-5p**	0.548626	1.39E-04	2	0.00233
**miR-22-3p**	0.536243	2.10E-04	3	0.00349
**miR-122-5p**	0.505436	0.000545917	4	0.00465
**miR-148a-3p**	0.485956	0.00095437	5	0.00581
**miR-378a-3p**	0.485503	0.000966472	6	0.00698
miR-532-5p	0.465157	0.002181351	7	0.00814
miR-320a	0.453186	0.002274844	8	0.00930
miR-486-5p	−0.45319	0.002274844	9	0.01047
**miR-21-5p**	0.437632	0.003337895	10	0.01163
**miR-101-3p**	0.413168	0.005892309	11	0.01279
miR-423-5p	0.340985	0.02524517	12	0.01395
**miR-30d-5p**	0.318571	0.03733837	13	0.01512
**miR-375**	0.342017	0.04432343	14	0.01628
miR-10b-5p	0.266687	0.08386032	15	0.01744

*The top 15 performing miRNAs are shown. miRNAs significantly correlating with disease severity (as by sequential organ failure assessment) are highlighted in bold black (SIRS) or red (sepsis). Refer to Table S7 in Supplementary Material for the full dataset*.

*^a^Green-shadowed cells indicate miRNAs that passed the BH correction for multiple comparisons*.

*^b^Blue- and violet-shadowed cells indicate miRNAs that returned positive and negative correlations (ρ ≥ 0.2 or ρ ≤ −0.2), respectively*.

*^c^Brown- and red-shadowed cells indicate miRNAs that returned significant correlations (p ≤ 0.05) and additionally passed the correction for multiple comparisons, respectively*.

## Results

### Severity of Non-Infective SIRS Positively Correlates with the Levels of CIR-miRNAs

**Figure 1:**
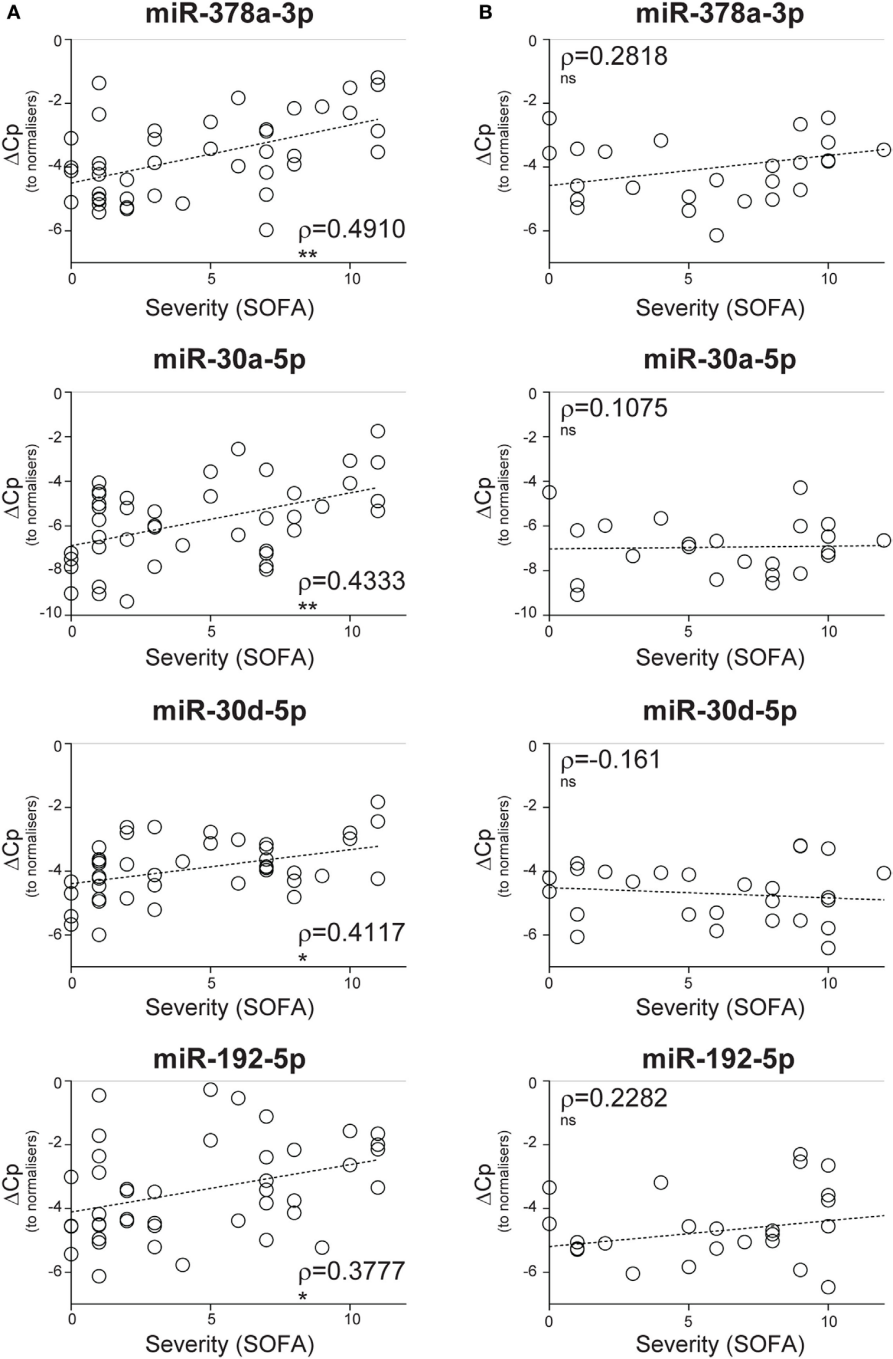
Circulating inflammatory-relevant microRNA levels correlate with the severity of non-infective systemic inflammatory response syndrome (SIRS). In miRNA qPCR arrays, within each patient’s specimen, crossing point (Cp) of a single miRNA is compared to the mean Cp of 2 normalizers (miR-486-5p and miR-320a) to give delta Cp (dCp). dCp of non-infective SIRS **(A)** and sepsis **(B)** patients were analyzed in correlation analyses with disease severity, as determined by the sequential organ failure assessment (SOFA) score. **(A)** Non-parametric correlation of SOFA scores with the plasma levels of miR-378a-3p, miR-30a-5p, miR-30d-5p, and miR-192-5p in non-infective SIRS patients (*n* = 43 for all miRNAs). **(B)** Non-parametric correlation of SOFA scores with the plasma levels of miR-378a-3p (*n* = 26), miR-30a-5p (*n* = 24), miR-30d-5p (*n* = 27), and miR-192-5p (*n* = 27) in infective SIRS (sepsis) patients. Each symbol represents an individual patient. Correlation trends are shown with the linear regression model including Spearman rho (ρ) and the significances of the correlations (*p ≤ 0.05; **p ≤ 0.005; and ***p ≤ 0.0005 or ns, non-significant).

To determine whether CIR-miRNA levels are regulated by the severity of SIRS, Cp of single miRNAs were compared to the mean Cp of internal normalizers [as previously identified (35)] to give dCp values that increase with the abundancy of specific miRNAs. Severity of disease, according to the SOFA score, was then correlated with dCp in non-infective SIRS patients. Thirteen CIR-miRNAs showed generally positive trends correlating with SIRS severity (Table 1). Significantly, 8 miRNAs showed a positive correlation with the disease severity (Table 1, black bold), with miR-378a-3p, miR-30a-5p, miR-30d-5p, and miR-192-5p further passing the Benjamini–Hochberg correction for multiple comparisons (52) (Figure 1A). We further determined whether CIR-miRNA levels correlate with disease severity in sepsis patients (Figure 1B). None of the CIR-miRNAs correlating with disease severity in SIRS showed similar significant trends in sepsis (Figure 1B; Table 2). Although miR-22-3p, miR-375, miR-122-5p, miR-192-5p, and miR-378a-3p maintained modestly positive non-significant trends, multiple CIR-miRNAs showed generally inverse trends with sepsis severity (Table 2).

**Figure 2:**
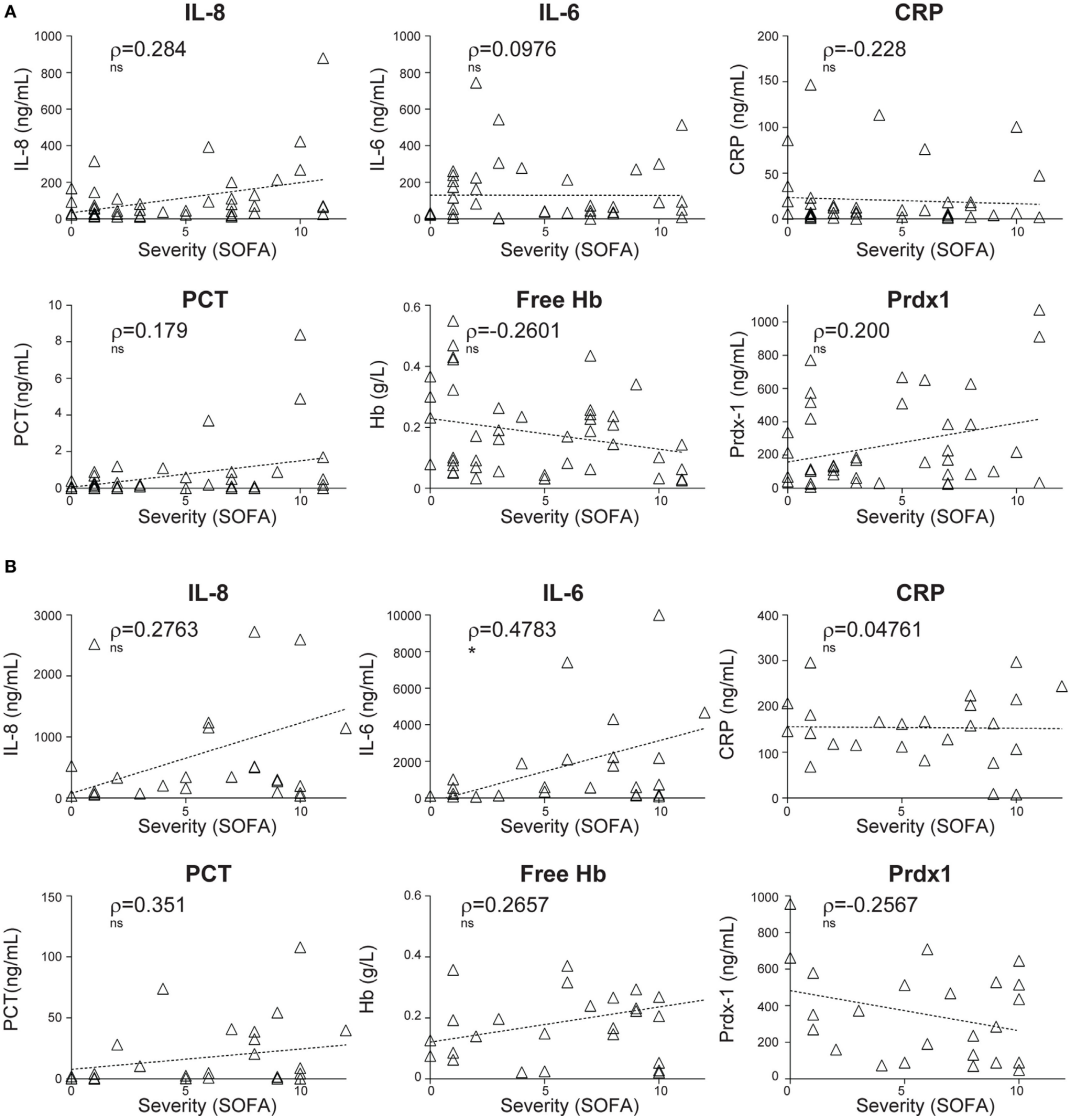
Correlations of plasma levels of inflammatory cytokines and stress mediators with the severity of non-infective systemic inflammatory response syndrome and sepsis. The levels of inflammatory cytokines (interleukin-IL-8 and IL-6) and stress mediators: C-reactive protein (CRP), pro-calcitonin (PCT), free hemoglobin (Hb), and peroxiredoxin-1 (Prdx-1) were measured by ELISA in the plasma of non-infective SIRS **(A)** and sepsis **(B)** patients. Thereafter, the correlation with the severity of disease, as determined by the sequential organ failure assessment (SOFA) score was investigated. **(A)** Non-parametric correlation of SOFA scores with the plasma levels of IL-8 (*n* = 43), IL-6 (*n* = 43), CRP (*n* = 43), PCT (*n* = 42), free Hb (*n* = 43), and Prdx-1 (*n* = 41) in non-infective SIRS patients. **(B)** Non-parametric correlation of SOFA scores with the plasma levels of IL-8 (*n* = 26), IL-6 (*n* = 27), CRP (*n* = 27), PCT (*n* = 27), free Hb (*n* = 27), and Prdx-1 (*n* = 24) in sepsis patients. Each triangle represents an individual patient. Correlation trends are shown with the linear regression model, including Spearman rho (ρ) and the significances of the correlations (* *p* ≤ 0.05; **p ≤ 0.005; and ***p ≤ 0.0005 or ns, non-significant).

In comparison to pro-inflammatory cytokines (IL-8 and IL-6) and soluble mediators of inflammation and stress such as CRP, PCT, free hemoglobin (Hb), and Prdx-1, CIR-miRNAs showed more robust correlations with disease severity (compare Figures 1A and 2A). None of these inflammatory mediators showed a significant correlation with SIRS severity, although some non-significant trends were apparent (Figure 2A). While levels IL-8, IL-6, CRP, and PCT increased in sepsis compared to non-infective SIRS, only IL-6 positively correlated with sepsis severity (Figure 2B).

Similar analyses were performed using the APACHE II in alternative to the SOFA severity score. In agreement with our previous analysis, CIR-miRNA levels positively and significantly correlated with the APACHE II score in non-infective SIRS (Figure S1A and Table S1 in Supplementary Material), but not in sepsis (Figure S1B and Table S2 in Supplementary Material). In addition, CIR-miRNAs in comparison to IL-6, CRP, PCT, and free Hb, generally showed more robust correlations with the APACHE II score, while IL-8 and Prdx-1 had significant positive correlation with the APACHE II score in non-infective SIRS (Figure S2A in Supplementary Material), but not in sepsis (Figure S2B in Supplementary Material).

### CIR-miRNAs Significantly Discriminate Severe from Non-Severe SIRS

**Figure 3:**
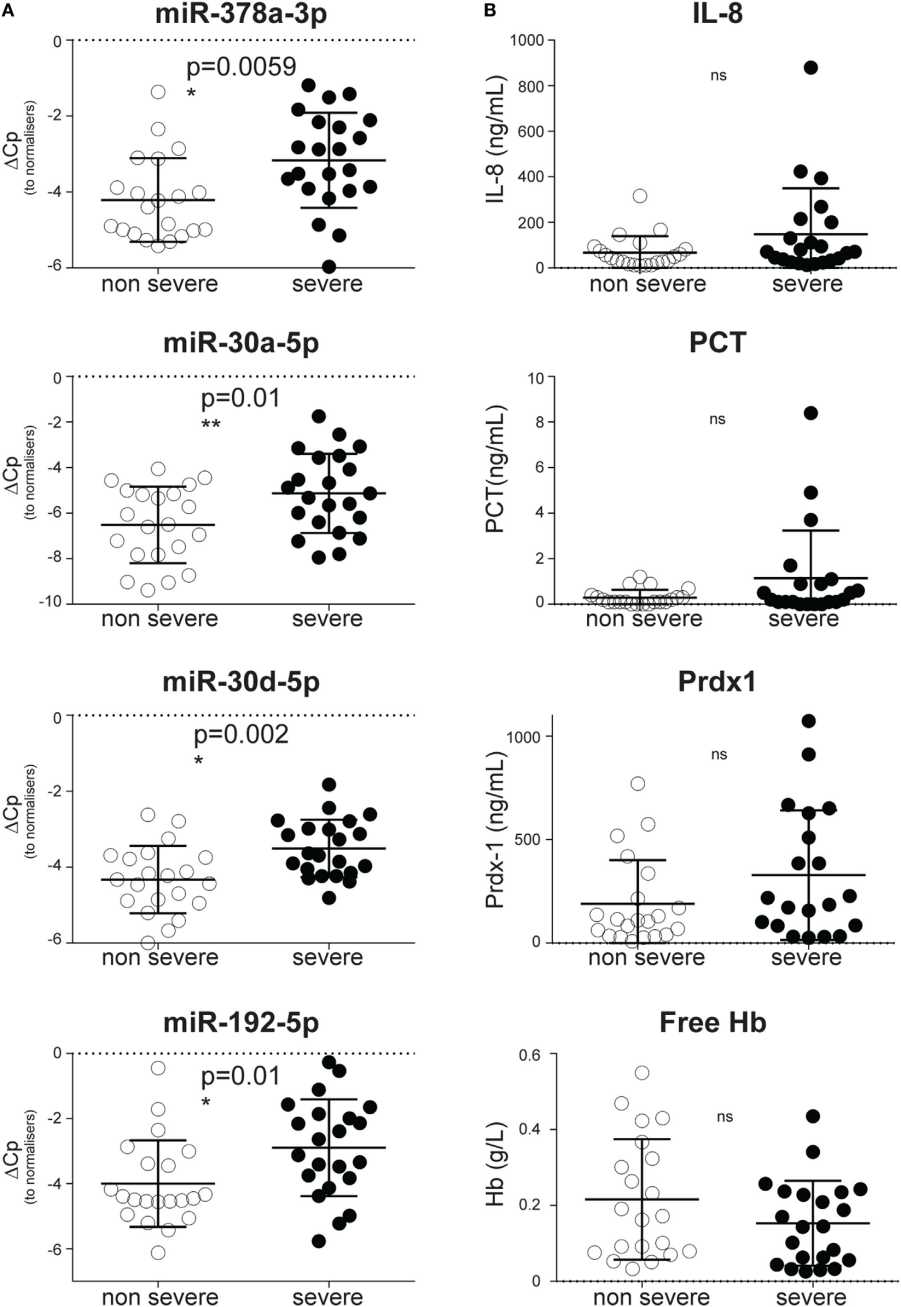
Circulating inflammatory-relevant microRNA biomarkers discriminate the severity of systemic inflammatory response syndrome (SIRS) better than inflammatory and stress mediators. In miRNA qPCR arrays, within each patient’s specimen, crossing point (Cp) of individual miRNAs were normalized as in Figure 1 and analyzed in patients with non-severe (open circles) and severe (filled circles) non-infective SIRS. Each symbol represents an individual patient. **(A)** Dot plots show delta Cp values for miR-378a-3p, miR-30a-5p, miR-30d-5p, and miR-192-5p in non-severe (*n* = 21) and severe (*n* = 22) non-infective SIRS patients, together with the level of significance. **(B)** Dot plots show concentration of IL-8, pro-calcitonin (PCT), peroxiredoxin-1 (Prdx-1) and free hemoglobin (Hb) in non-severe (*n* = 21) and severe (*n* = 22, apart from Prdx-1 in which *n* = 20) non-infectious SIRS patients, together with the level of significance.

Consistent with a steady accumulation of CIR-miRNAs in more severe disease, dCp values were higher in severe rather than non-severe SIRS (Figure 3A) for the most significantly affected CIR-miRNAs. Hence, miR-378a-3p, miR-30a-5p, miR-30d-5p, and miR-192-5p distinguished non-severe from severe SIRS patients (Figure 3A). Also miR-122-5p, miR-101-3p, miR-21-5p, miR-148a-3p, and miR-532-5p (which all had non-significant positive trends to increase with SIRS severity, Table 1) significantly discriminated severe from non-severe SIRS (Figure S3 in Supplementary Material). By contrast, none of the inflammatory cytokines and stress mediators we measured in our cohort discriminated SIRS severity groups, including IL-8 (Figure 3B) and IL-6 (Figure S3B in Supplementary Material).

### SIRS-Relevant CIR-miRNAs Are Unlikely to Derive from RBCs

**Figure 4:**
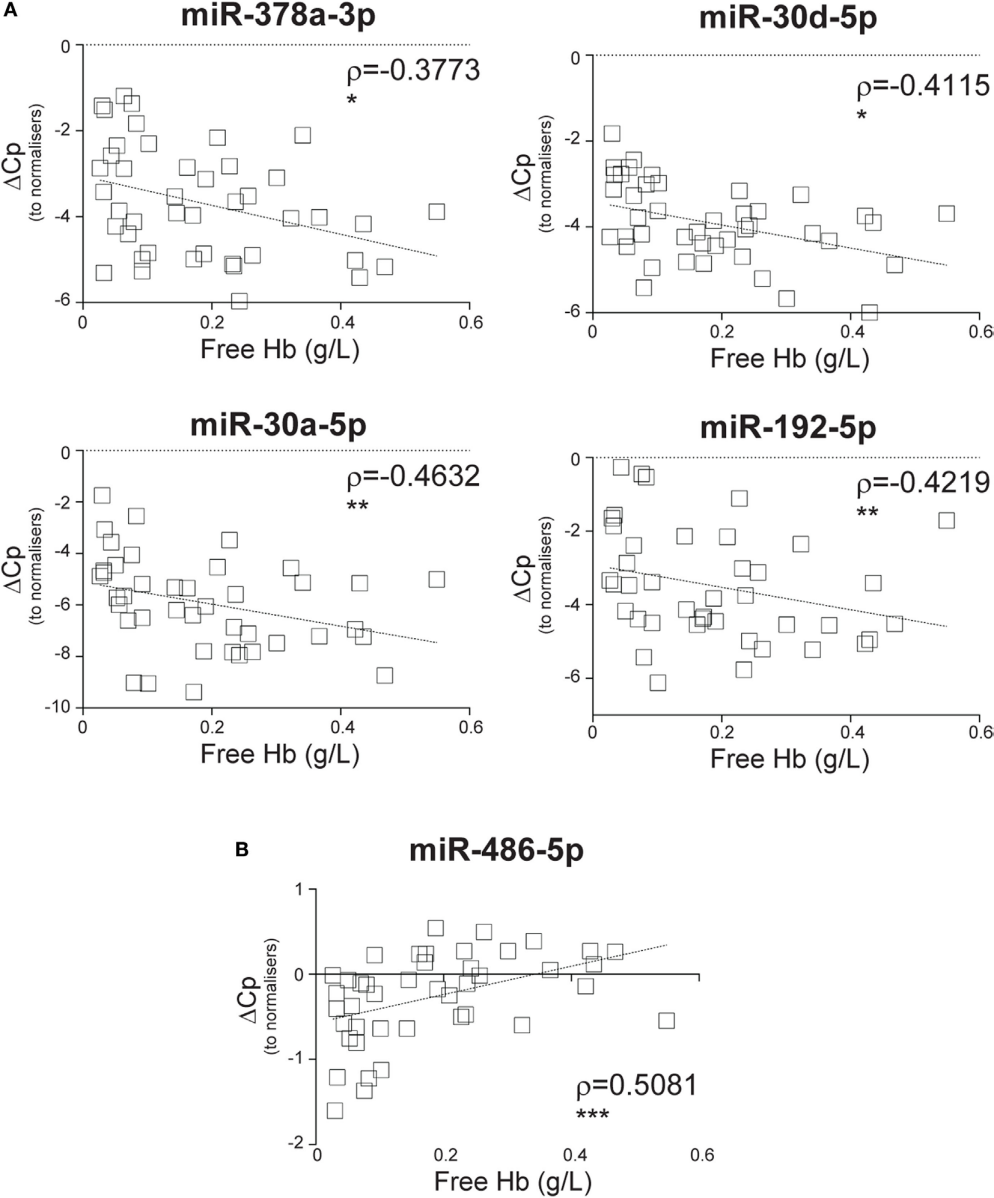
Circulating inflammatory-relevant microRNA levels inversely correlate with markers of red-blood cell lysis. In miRNA qPCR arrays, within each patient’s specimen, crossing point (Cp) of individual miRNAs (open squares) were normalized as in Figure 1 and analyzed in correlation with levels of free Hb, which is derived from the lysis of red blood cells. Each square represents an individual patient. Correlation trends are shown with the linear regression model including Spearman rho (ρ) and the significances of the correlations (*p ≤ 0.05; **p ≤ 0.005; and ***p ≤ 0.0005 or ns, non-significant) for **(A)** miR-378a-3p, miR-30a-5p, miR-30d-5p, and miR-192-5p and **(B)** miR-486-5p in non-infective systemic inflammatory response syndrome patients (*n* = 43).

We next investigated where the CIR-miRNAs regulated by SIRS severity may originate. We reasoned that if they derived from RBCs that contain multiple miRNAs (released in the blood after physiological hemolysis or coagulopathy), then CIR-miRNA levels would positively correlate with the amount of free Hb. Instead, around 95% of the analyzed CIR-miRNAs (40/43) showed inverse correlation trends with free Hb (Tables 3; Table S6 in Supplementary Material). Many CIR-miRNAs, including miR-378a-3p, miR-30a-5p, miR-30d-5p, and miR-192-5p (Figure 4A) and miR-101-3p, miR-21-5p, miR-22-3p, miR-423-5p, and miR-122-5p (Figure S4 in Supplementary Material) significantly inversely correlated with free Hb levels even after a stringent Benjamini–Hochberg correction (52), supporting that CIR-miRNAs relevant in severe SIRS do not derive from RBCs. By contrast, free Hb levels positively correlated with miRNAs abundant in RBCs (29), miR-486-5p and miR-451a (Figure 4B; Table 3), consistent with a RBC origin.

### CIR-miRNAs Positively Correlate with Redox Biomarker Prdx-1

**Figure 5:**
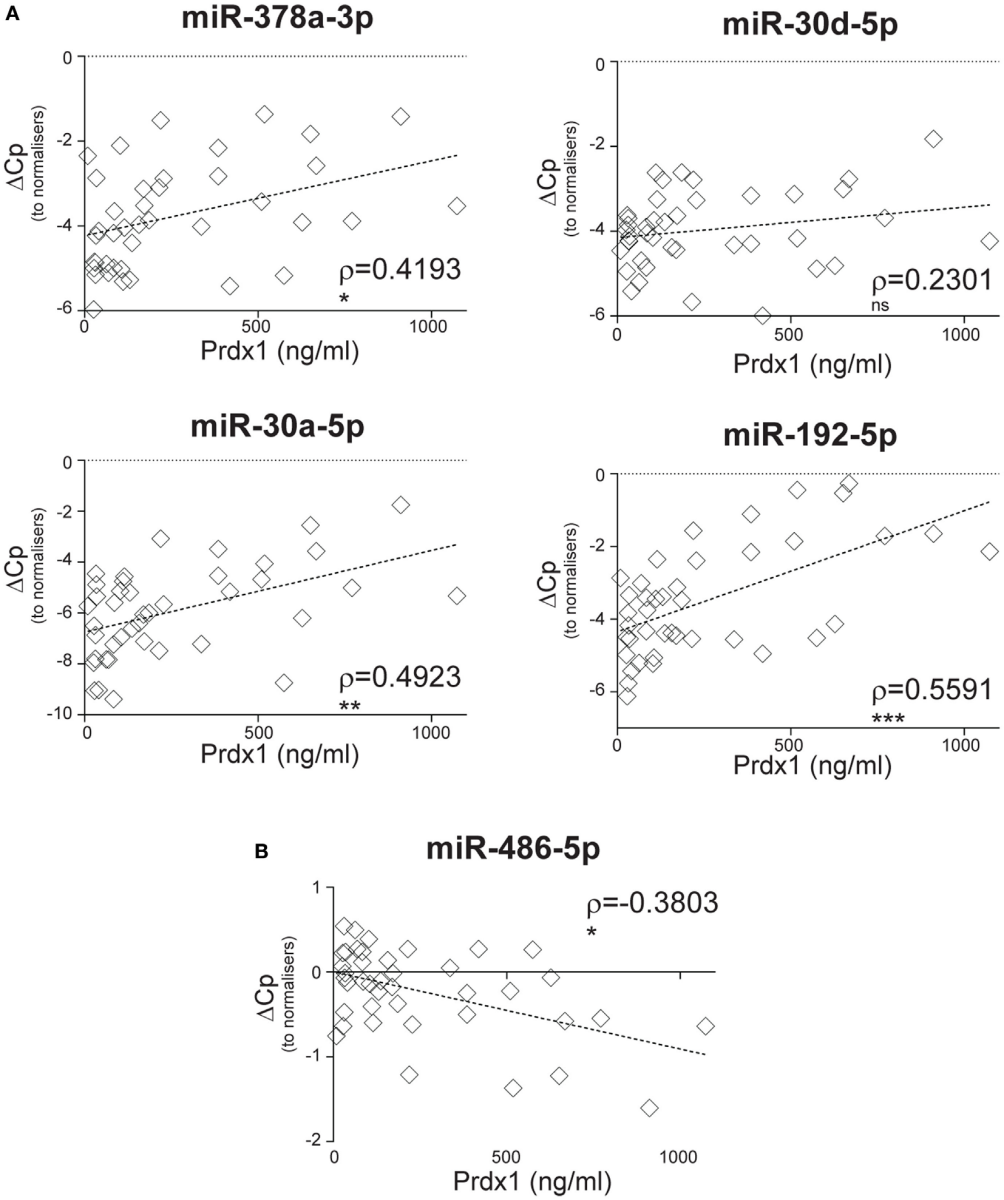
Circulating inflammatory-relevant microRNA levels positively correlate with markers of immunological stress, peroxiredoxin-1 (Prdx1). In miRNA qPCR arrays, within each patient’s specimen, crossing point (Cp) of individual microRNAs (miRNAs) (open rhombi) were normalized as in Figure 1 and analyzed in correlation with levels of plasma inflammatory stress marker, Prdx-1. Each symbol represents an individual patient. Correlation trends are shown with the linear regression model including Spearman rho (ρ) and the significances of the correlations (*p ≤ 0.05; **p ≤ 0.005; and ***p ≤ 0.0005 or ns, non-significant) for **(A)** miR-378a-3p, miR-30a-5p, miR-30d-5p, and miR-192-5p, and **(B)** miR-486-5p in non-infective systemic inflammatory response syndrome patients (*n* = 41).

We sought to investigate whether SIRS-relevant CIR-miRNAs derive from immune cells (i.e., white blood cells). To do this, we used Prdx-1, a marker that we had previously found to be released by immune cells after inflammatory stimuli (36, 37). Consistently, in the entire SIRS cohort, Prdx-1 levels correlated with markers of immune activation (IL-8 and sCD25), rather than free Hb (Figure S5 in Supplementary Material), demonstrating that Prdx-1 did not increase due to RBC hemolysis. We then analyzed the correlation of Prdx-1 with CIR-miRNA dCp (Figure 5; Table 4). Around 40% (18/43) of CIR-miRNAs showed moderate-to-strong correlation trends with Prdx-1 levels, with a prevalence of positive relationships (Tables 4; Table S7 in Supplementary Material). Levels of 10 CIR-miRNAs, including miR-378a-3p, miR-30a-5p, and miR-192-5p (Figure 5A) and miR-101-3p, miR-21-5p, miR-22-3p, and miR-122-5p (Figure S6 in Supplementary Material) significantly positively correlated with those of Prdx-1, suggesting that the amounts of CIR-miRNAs modulated in non-infective SIRS mirror those of this inflammatory biomarker. By contrast, miRNAs abundant in RBCs, miR-486-5p (Figure 5B) and miR-451a (Table S7 in Supplementary Material), did not show positive correlation with Prdx-1.

### Stimulated Immune Cells Produce CIR-miRNAs Affected by Severity of SIRS

**Figure 6:**
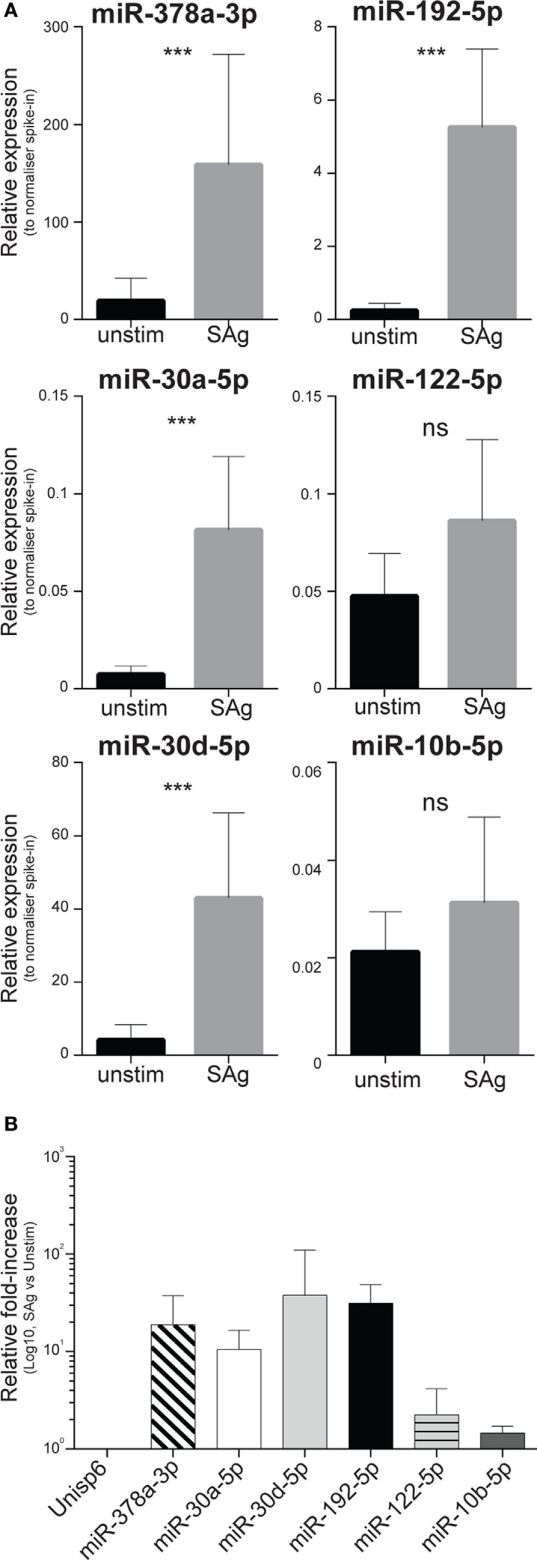
Levels of circulating inflammatory-relevant microRNAs affected by the severity of systemic inflammatory response syndrome (SIRS) are increased in supernatants of blood-derived immune cell cultures. PBMCs derived from 10 individuals were freshly purified from blood and equal cell numbers were then incubated in complete media in the presence of exosome-free bovine serum, strictly at the concentration of 2 × 10^6^ cells/ml, in replicate wells. Half of the cultures were stimulated with the SPE-KL bacterial superantigen (SAg) from 5 days, in comparison to unstimulated controls (unstim). On day 5, cells were harvested and equal volumes of supernatants were recovered after two sequential spins prior to RNA extraction as detailed in the Section “Materials and Methods.” The presence of microRNAs regulated (miR-378a-3p, miR-30a-5p, miR-30d-5p, miR-192-5p, and miR-122-5p) or unaffected (miR-10b-5p) by the severity of non-infective SIRS was assessed in Q-PCR array in multiple biological replicates. **(A)** Within each donor’s specimen, crossing point (Cp) of a single miRNA is compared to the Cp of a spike-in RNA added to supernatants just prior to the RNA-purification to give delta Cp (dCp). dCp were then linearized to give the relative expression of individual miRNAs in supernatants from unstimulated (black bars; *n* = 10, 7, 5, 7, 10, 4, respectively, for miR-378a-3p, miR-30a-5p, miR-30d-5p, miR-192-5p, miR-122-5p, and miR-10b-5p) compared to SAg-activated cells (gray bars; *n* = 10, 10, 8, 9, 10, and 9, respectively, for miR-378a-3p, miR-30a-5p, miR-30d-5p, miR-192-5p, miR-122-5p, and miR-10b-5p). **(B)** Average miRNA fold-induction in 4–10 individuals were calculated as the average ratio of miRNA levels detected in supernatants from SAg-activated cells compared to unstimulated cultures.

To determine whether immune cells could produce miR-378a-3p, miR-30a-5p, miR-30d-5p, and miR-192-5p, PBMCs of 10 healthy donors were stimulated *in vitro* with bacterial SAg, known to drive massive inflammatory cell activation. After 5 days, viability of the cell cultures was determined using the Trypan-blue dye exclusion assay (Figure S7 in Supplementary Material). Relative to a normalizer spike-in, we found increased amounts of miR-378a-3p, miR-30a-5p, miR-30d-5p, and miR-192-5p in culture supernatants of stimulated PBMCs compared to unstimulated controls (Figure 6A). The increase of miRNAs in the supernatants varied across the miRNA species between 10- and 100-fold (Figure 6B), with the notable exception of miR-122-5p that was not significantly increased (Figures 6A,B). In cultures derived from four donors, levels of miR-10b-5p, which did not discriminate non-severe from severe SIRS (Figure S3 in Supplementary Material), did not increase upon activation (Figures 6A,B).

## Discussion

We report here that, during non-infective SIRS, the blood levels of CIR-miRNAs increase in parallel with the severity of inflammation. Levels of CIR-miRNAs do not correlate with those of free Hb, indicating that they do not derive from RBCs during SIRS. Instead, CIR-miRNAs positively correlate with levels of the inflammatory mediator and redox enzyme, Prdx-1 which is released by immune cells in inflammation. Consistently miR-378a-3p, miR-30a-5p, miR-30d-5p, and miR-192-5p affected by severity of SIRS are produced by immune cells upon activation. As CIR-miRNAs are increasingly proposed as biomarkers for sepsis, cancer, and other disease (35, 53–55), their inflammatory cell origin should be considered in future research.

In our study, CIR-miRNAs distinguish non-severe from severe SIRS better than inflammatory cytokines and mediators. Inflammatory cytokines are thought to be released during SIRS as part of the cytokine storm, during MODS (1). As in our current study, plasma levels of cytokines have not previously been found to reliably increase in severe SIRS (1), perhaps due to low cytokine concentration and short half-life in samples (56, 57). By contrast, regulators of inflammatory mediators are abundant in the blood of SIRS patients (20), as we found to be the case for the CIR-miRNAs affected by SIRS severity (Figure 3; Figure S3 in Supplementary Material). In addition, it should be noted that blood levels of miR-378a-3p, miR-30a-5p, miR-30d-5p, and miR-192-5p were regulated by disease severity, irrespectively from whether patients were taking anti-inflammatory drugs (at the time of admission) (Figure S8 and Table S8 in Supplementary Material).

CIR-miRNAs may act as regulators of inflammation (35) by targeting the 3′UTR of mRNAs encoding pro-inflammatory cytokines/mediators (58–61). Many miRNAs are involved in inflammation/immune function (27), including miR-378 (62, 63), miR-30a/d (64, 65), miR-192 (64–68), and others found in this study (69). Interestingly, the more severe SIRS is, the more CIR-miRNAs are released, potentially counteracting systemic inflammation as part of the CAR (24). Significantly, direct or indirect targets of miR-378, miR-30a/d, and miR-192 include, among other genes, IL-1A (70), IL-1R (71), IL-17 (72), IL-17RA, and IL-17RE (73). In contrast, CIR-miRNA levels did not correlate with sepsis severity (compare Tables 1 and 2; Tables S1 and S2 in Supplementary Material), suggesting that inflammatory pathways leading to CIR-miRNA accumulation in blood are dysregulated in sepsis. Consequently, upon sepsis progression, the negative regulation exerted by CIR-miRNAs may be restrained, thus boosting immunopathology. Inflammatory cytokine (IL-1, IL-6, etc.) levels increased consistently much more in sepsis than in SIRS (35), therefore inflammatory mRNAs may act as a “sponge” to sequestrate miRNAs in sepsis, but not in other trauma including SIRS (74). Although miR-30a was recently associated with induction of myeloid derived suppressor cells in cancer (75), the question of whether CIR-miRNAs are anti-inflammatory and ameliorate sepsis outcome remains to be further investigated.

Alternatively, CIR-miRNAs may act as modulators of inflammation *via* indirect effects (i.e., by not targeting directly inflammatory cytokine expression). For instance, at least in mice, miR-378 is known to target phosphoinositide 3 kinase (PI3K) expression in liver cells, affecting liver metabolism with systemic effects which may be relevant during systemic inflammatory disease and MODS (76). Furthermore, PI3K pathway is also crucially regulated during immune cell proliferation, differentiation, and apoptosis (77), all of which may affect immune cell function during SIRS. Similarly, miR-30a has been shown to target the expression of Blimp-1 (78) (an important differentiation factor in immune cells) and various members of the calcineurin signaling pathway, including NFATc3 (79) at least in podocytes and cardiomyocytes, which might have implications for MODS and inflammation. Finally, miR-192 has been shown to suppress cell-proliferation pathways in myeloma (80) and other leukemic cells (81). However for all abovementioned cases, whether the same targets are also regulated in normal immune cells remains so far elusive.

Altogether, our results have implications for clinical practice and future therapeutic interventions. In particular in ICU/HDU, using miRNAs to distinguish non-infective SIRS from sepsis and severe from non-severe SIRS could help guide patient management, for example, triaging patients based on severity, informing decisions based on prognosis, and helping target therapy including the need for and choice of antibiotics. In future, miRNAs may become markers and/or targets for novel immunotherapy for acute inflammatory conditions, potentially providing a non-antibiotic alternative intervention.

The origin of circulating miRNAs is unclear. RBCs contain miRNAs that they release upon hemolysis (39, 40). To exclude artifacts from sample processing, we removed hemolytic samples and used plasma rather than serum, as the latter contains more Hb (and RBC miRNAs) due to coagulation damage (82, 83). Coagulopathy may drive pathophysiological levels of hemolysis and release of CIR-miRNAs, as seen for miR-486-5p and miR-451a [abundant in RBCs (29)]. Although free Hb levels did not significantly increase with SIRS severity, levels of multiple CIR-miRNAs inversely correlate with free Hb, indicating that these do not derive from RBCs in SIRS as in sepsis.

Our data suggest that CIR-miRNAs affected in SIRS may derive from inflammatory cells activated during disease. In particular, CIR-miRNAs may be released in the blood in association with inflammation-induced oxidative stress, as suggested by the positive correlation between Prdx-1 and multiple CIR-miRNAs. Prdx-1 is an anti-oxidant enzyme that mediates the elimination of H_2_O_2_ and can be secreted as a dimer by macrophages, upon TLR triggering (36, 37). Its expression is regulated mainly by the transcription factor Nrf2, activated by ROS and various other reactive, electrophilic species (84). Its induction may be an indicator of a protective response to oxidative stress (85, 86). Because Prdx-1 is associated with immune cells activation (37), we asked whether SIRS-relevant CIR-miRNAs were derived from circulating immune cells upon activation. We found that miRNAs affected by SIRS severity were indeed increased >10-fold in supernatants of PBMC cultures after activation with bacterial SAg that drove ~1.5-fold cell-expansion. Thus, sustained immune cell activation may explain the massive release of miRNAs regulated during severe SIRS. Macrophages/monocytes, T and NK cells could be possible candidates for the release of CIR-miRNAs.

Of note, activated PBMCs did not produce significant increase in the supernatant levels of miR-10b-5p, which was not affected by SIRS severity (Figure S3 in Supplementary Material). Unlike other miRNAs, the modest (<2-fold) increase of miR-122-5p upon SAg stimulation may be explained simply by cell proliferation, suggesting that PBMCs contribute little to miR-122-5p blood levels. As hepatocytes highly express miR-122 and increased blood levels of miR-122 were previously associated with liver pathology (87), epithelial cell release of miR-122 and potentially other CIR-miRNAs during SIRS should be further investigated in future.

In conclusion, our work opens up a number of questions about CIR-miRNAs for future investigation. Currently, the exact function of circulating miRNAs in inflammatory conditions remains unknown. CIR-miRNAs released by cells in the body after inflammatory damage may act as DAMPs that bind to TLR ligands (33), a property that is shared by Prdx-1 (86). For instance, small RNAs contained in exosomes can be phagocytosed in myeloid-origin, antigen-presenting like cells and trigger TLR7 signaling, *in vitro* (31). In this context, miRNAs may act as pro-inflammatory DAMPs to drive inflammatory cytokines (including IL-6) production downstream of intracellular TLRs (31). However, we previously found that pro-inflammatory cytokine levels inversely correlate with blood levels of CIR-miRNAs, suggesting that CIR-miRNAs may negatively regulate inflammation *in vivo* (35). In mice, regulatory T cells (Tregs) have been shown to dampen the activity of conventional T cells by miRNA transfer (88). Yet, it is unclear whether miRNAs from Tregs can exert suppression at distance. Furthermore, beyond targeting cytokine and inflammatory mediator expression, CIR-miRNAs may regulate inflammation also *via* indirect mechanisms, or a complex combination of direct and indirect mechanisms, by acting in concert on distinct targets, in multiple cell types, and in different organs. The dynamic contribution of immune cells to blood miRNAs has been frequently overlooked, despite inflammation clearly affecting them. Thus, while differences in miRNA transfer may exist *in vivo* compared to *in vitro*, future research is needed to clarify the role of CIR-miRNAs in immunopathological and homeostatic conditions. In particular, more research is needed to address which immune cells specifically produce CIR-miRNAs to be released in the blood, including when and how. Also the mode of trafficking and the cellular and molecular targets of CIR-miRNAs need to be identified yet. In future, such research may allow to harness CIR-miRNAs as immunomodulatory drugs useful for therapeutic purposes in inflammatory conditions as in other diseases.

## Ethics Statement

Written informed consent or consultee approval to enroll was secured for all study participants (patients and healthy donors). This study was approved by the North Wales Research Ethics Committee (Central and East, reference 10/WNo03/19) and the BSMS Research Governance and Ethics Committee (reference: 13/182/LLE) and carried out in accordance with the approved guidelines. All data were anonymized.

## Author Contributions

SC and MM performed experiments and analyzed the data; SC, PG, SN, FK, and ML conceived and designed experiments; SC, SN, and ML wrote the first manuscript draft. All authors critically contributed to the final version of the manuscript.

## Conflict of Interest Statement

The authors have no conflict of interest directly related to the current manuscript. However, SC, FK, SN, and ML are inventors in a patent submitted by the University of Sussex for the use of CIR-miRNAs as biomarkers of sepsis.

## Abbreviations

miRNA, microRNA; CIR-miRNA, circulating inflammatory-relevant microRNA; SIRS, systemic inflammatory response syndrome; SOFA, sequential organ failure assessment; APACHE II, acute physiology and chronic health evaluation II; MODS, multiple organ dysfunction syndrome; CAR, compensatory anti-inflammatory responses; DAMPs, damage-associated molecular pattern molecules; PAMPs, pathogen-associated molecular pattern molecules; TLR, toll-like receptor; ROS, reactive oxygen species; TNF, tumor necrosis factor; TGF, tumor growth factor; CRP, C-reactive protein; sCD25, soluble CD25; Prdx-1, peroxiredoxin-1; Hb, hemoglobin; IL-, interleukin; PCT, pro-calcitonin; SAg, superantigen; SPE-K/L, streptococcal pyrogenic exotoxin K/L; PBMCs, peripheral blood mononuclear cells; RBCs, red blood cells; PBS, phosphate-buffered saline; FCS, fetal calf serum; RPMI, Roswell Park Memorial Institute medium; RT, reverse transcription/transcribed; IQR, interquartile ranges.

## References

[1] Davies MG Hagen PO. Systemic inflammatory response syndrome. Br J Surg (1997) 84(7):920–35. 10.1002/bjs.1800840707 9240130

[2] Singer M Deutschman CS Seymour CW Shankar-Hari M Annane D Bauer M The third international consensus definitions for sepsis and septic shock (sepsis-3). JAMA (2016) 315(8):801–10. 10.1001/jama.2016.0287 26903338 PMC4968574

[3] Brun-Buisson C. The epidemiology of the systemic inflammatory response. Intensive Care Med (2000) 26(Suppl 1):S64–74. 10.1007/s001340051121 10786961 PMC7094973

[4] Vincent JL de Mendonca A Cantraine F Moreno R Takala J Suter PM Use of the SOFA score to assess the incidence of organ dysfunction/failure in intensive care units: results of a multicenter, prospective study. Working group on “sepsis-related problems” of the European Society of Intensive Care Medicine. Crit Care Med (1998) 26(11):1793–800. 10.1097/00003246-199811000-00016 9824069

[5] Knaus WA Draper EA Wagner DP Zimmerman JE. APACHE II: a severity of disease classification system. Crit Care Med (1985) 13(10):818–29. 10.1097/00003246-198510000-00009 3928249

[6] Matsuda N Hattori Y Systemic inflammatory response syndrome (SIRS): molecular pathophysiology and gene therapy. J Pharmacol Sci (2006) 101(3):189–98. 10.1254/jphs.CRJ06010X 16823257

[7] Cohen J The immunopathogenesis of sepsis. Nature (2002) 420(6917):885–91. 10.1038/nature01326 12490963

[8] Johnson GB Brunn GJ Platt JL. Cutting edge: an endogenous pathway to systemic inflammatory response syndrome (SIRS)-like reactions through toll-like receptor 4. J Immunol (2004) 172(1):20–4. 10.4049/jimmunol.172.1.20 14688304

[9] Makhija R Kingsnorth AN. Cytokine storm in acute pancreatitis. J Hepatobiliary Pancreat Surg (2002) 9(4):401–10. 10.1007/s005340200049 12483260

[10] Ulloa L Tracey KJ The “cytokine profile”: a code for sepsis. Trends Mol Med (2005) 11(2):56–63. 10.1016/j.molmed.2004.12.007 15694867

[11] Kietzmann D Kahl R Muller M Burchardi H Kettler D Hydrogen peroxide in expired breath condensate of patients with acute respiratory failure and with ARDS. Intensive Care Med (1993) 19(2):78–81. 10.1007/BF01708366 8486874

[12] Quinlan GJ Evans TW Gutteridge JM. 4-hydroxy-2-nonenal levels increase in the plasma of patients with adult respiratory distress syndrome as linoleic acid appears to fall. Free Radic Res (1994) 21(2):95–106. 10.3109/10715769409056561 7921168

[13] Nussler AK Billiar TR. Inflammation, immunoregulation, and inducible nitric oxide synthase. J Leukoc Biol (1993) 54(2):171–8. 7689630

[14] Metnitz PG Bartens C Fischer M Fridrich P Steltzer H Druml W. Antioxidant status in patients with acute respiratory distress syndrome. Intensive Care Med (1999) 25(2):180–5. 10.1007/s001340050813 10193545

[15] Gutteridge JM Mitchell J. Redox imbalance in the critically ill. Br Med Bull (1999) 55(1):49–75. 10.1258/0007142991902295 10695079

[16] Swank DW Moore SB. Roles of the neutrophil and other mediators in adult respiratory distress syndrome. Mayo Clin Proc (1989) 64(9):1118–32. 10.1016/S0025-6196(12)64981-7 2682051

[17] Botha AJ Moore FA Moore EE Kim FJ Banerjee A Peterson VM. Postinjury neutrophil priming and activation: an early vulnerable window. Surgery (1995) 118(2):358–64; discussion 64–5. 10.1016/S0039-6060(05)80345-9 7638753

[18] Botha AJ Moore FA Moore EE Sauaia A Banerjee A Peterson VM. Early neutrophil sequestration after injury: a pathogenic mechanism for multiple organ failure. J Trauma (1995) 39(3):411–7. 10.1097/00005373-199509000-00003 7473901

[19] Gando S Kameue T Nanzaki S Nakanishi Y. Disseminated intravascular coagulation is a frequent complication of systemic inflammatory response syndrome. Thromb Haemost (1996) 75(2):224–8. 8815564

[20] Goldie AS Fearon KC Ross JA Barclay GR Jackson RE Grant IS Natural cytokine antagonists and endogenous antiendotoxin core antibodies in sepsis syndrome. The sepsis intervention group. JAMA (1995) 274(2):172–7. 10.1001/jama.1995.03530020090038 7596007

[21] Ferguson KL Taheri P Rodriguez J Tonapi V Cardellio A Dechert R. Tumor necrosis factor activity increases in the early response to trauma. Acad Emerg Med (1997) 4(11):1035–40. 10.1111/j.1553-2712.1997.tb03676.x 9383488

[22] Nast-Kolb D Waydhas C Gippner-Steppert C Schneider I Trupka A Ruchholtz S Indicators of the posttraumatic inflammatory response correlate with organ failure in patients with multiple injuries. J Trauma (1997) 42(3):446–54; discussion 54–5. 10.1097/00005373-199703000-00012 9095112

[23] Lyons A Kelly JL Rodrick ML Mannick JA Lederer JA. Major injury induces increased production of interleukin-10 by cells of the immune system with a negative impact on resistance to infection. Ann Surg (1997) 226(4):450–8; discussion 8–60. 10.1097/00000658-199710000-00006 9351713 PMC1191059

[24] Ward NS Casserly B Ayala A. The compensatory anti-inflammatory response syndrome (CARS) in critically ill patients. Clin Chest Med (2008) 29(4):617–25, viii. 10.1016/j.ccm.2008.06.010 18954697 PMC2786900

[25] Bartel DP. MicroRNAs: genomics, biogenesis, mechanism, and function. Cell (2004) 116(2):281–97. 10.1016/S0092-8674(04)00045-5 14744438

[26] Bartel DP MicroRNAs: target recognition and regulatory functions. Cell (2009) 136(2):215–33. 10.1016/j.cell.2009.01.002 19167326 PMC3794896

[27] Kroesen BJ Teteloshvili N Smigielska-Czepiel K Brouwer E Boots AM van den Berg A Immuno-miRs: critical regulators of T-cell development, function and ageing. Immunology (2015) 144(1):1–10. 10.1111/imm.12367 25093579 PMC4264905

[28] Arroyo JD Chevillet JR Kroh EM Ruf IK Pritchard CC Gibson DF Argonaute2 complexes carry a population of circulating microRNAs independent of vesicles in human plasma. Proc Natl Acad Sci U S A (2011) 108(12):5003–8. 10.1073/pnas.1019055108 21383194 PMC3064324

[29] Wang K Yuan Y Cho JH McClarty S Baxter D Galas DJ. Comparing the MicroRNA spectrum between serum and plasma. PLoS One (2012) 7(7):e41561. 10.1371/journal.pone.0041561 22859996 PMC3409228

[30] Duttagupta R Jiang R Gollub J Getts RC Jones KW. Impact of cellular miRNAs on circulating miRNA biomarker signatures. PLoS One (2011) 6(6):e20769. 10.1371/journal.pone.0020769 21698099 PMC3117799

[31] Fabbri M Paone A Calore F Galli R Gaudio E Santhanam R MicroRNAs bind to toll-like receptors to induce prometastatic inflammatory response. Proc Natl Acad Sci U S A (2012) 109(31):E2110–6. 10.1073/pnas.1209414109 22753494 PMC3412003

[32] Lehmann SM Kruger C Park B Derkow K Rosenberger K Baumgart J An unconventional role for miRNA: let-7 activates toll-like receptor 7 and causes neurodegeneration. Nat Neurosci (2012) 15(6):827–35. 10.1038/nn.3113 22610069

[33] Fabbri M. TLRs as miRNA receptors. Cancer Res (2012) 72(24):6333–7. 10.1158/0008-5472.CAN-12-3229 23222301

[34] Chen X Liang H Zhang J Zen K Zhang CY. MicroRNAs are ligands of toll-like receptors. RNA (2013) 19(6):737–9. 10.1261/rna.036319.112 23554231 PMC3683908

[35] Caserta S Kern F Cohen J Drage S Newbury SF Llewelyn MJ. Circulating plasma microRNAs can differentiate human sepsis and systemic inflammatory response syndrome (SIRS). Sci Rep (2016) 6:28006. 10.1038/srep28006 27320175 PMC4913253

[36] Checconi P Salzano S Bowler L Mullen L Mengozzi M Hanschmann EM Redox proteomics of the inflammatory secretome identifies a common set of redoxins and other glutathionylated proteins released in inflammation, influenza virus infection and oxidative stress. PLoS One (2015) 10(5):e0127086. 10.1371/journal.pone.0127086 25985305 PMC4436175

[37] Mullen L Hanschmann EM Lillig CH Herzenberg LA Ghezzi P. Cysteine oxidation targets peroxiredoxins 1 and 2 for exosomal release through a novel mechanism of redox-dependent secretion. Mol Med (2015) 21:98–108. 10.2119/molmed.2015.00033 25715249 PMC4461588

[38] Llewelyn MJ Berger M Gregory M Ramaiah R Taylor AL Curdt I Sepsis biomarkers in unselected patients on admission to intensive or high-dependency care. Crit Care (2013) 17(2):R60. 10.1186/cc12588 23531337 PMC3672658

[39] Pritchard CC Kroh E Wood B Arroyo JD Dougherty KJ Miyaji MM Blood cell origin of circulating microRNAs: a cautionary note for cancer biomarker studies. Cancer Prev Res (Phila) (2012) 5(3):492–7. 10.1158/1940-6207.CAPR-11-0370 22158052 PMC4186243

[40] Kirschner MB Kao SC Edelman JJ Armstrong NJ Vallely MP van Zandwijk N Haemolysis during sample preparation alters microRNA content of plasma. PLoS One (2011) 6(9):e24145. 10.1371/journal.pone.0024145 21909417 PMC3164711

[41] Harboe M A method for determination of hemoglobin in plasma by near-ultraviolet spectrophotometry. Scand J Clin Lab Invest (1959) 11:66–70. 10.3109/00365515909060410 13646603

[42] Adamzik M Hamburger T Petrat F Peters J de Groot H Hartmann M. Free hemoglobin concentration in severe sepsis: methods of measurement and prediction of outcome. Crit Care (2012) 16(4):R125. 10.1186/cc11425 22800762 PMC3580706

[43] Han V Serrano K Devine DV. A comparative study of common techniques used to measure haemolysis in stored red cell concentrates. Vox Sang (2010) 98(2):116–23. 10.1111/j.1423-0410.2009.01249.x 19719459

[44] Lippi G Salvagno GL Montagnana M Brocco G Guidi GC. Influence of hemolysis on routine clinical chemistry testing. Clin Chem Lab Med (2006) 44(3):311–6. 10.1515/CCLM.2006.054 16519604

[45] Blondal T Jensby Nielsen S Baker A Andreasen D Mouritzen P Wrang Teilum M Assessing sample and miRNA profile quality in serum and plasma or other biofluids. Methods (2013) 59(1):S1–6. 10.1016/j.ymeth.2012.09.015 23036329

[46] Caserta S Taylor AL Terrazzini N Llewelyn MJ Induction of human regulatory T cells with bacterial superantigens. Methods Mol Biol (2016) 1396:181–206. 10.1007/978-1-4939-3344-0_16 26676048

[47] Taylor AL Llewelyn MJ. Superantigen-induced proliferation of human CD4+CD25- T cells is followed by a switch to a functional regulatory phenotype. J Immunol (2010) 185(11):6591–8. 10.4049/jimmunol.1002416 21048104

[48] Andersen CL Jensen JL Orntoft TF. Normalization of real-time quantitative reverse transcription-PCR data: a model-based variance estimation approach to identify genes suited for normalization, applied to bladder and colon cancer data sets. Cancer Res (2004) 64(15):5245–50. 10.1158/0008-5472.CAN-04-0496 15289330

[49] Cohen J Statistical Power Analysis for the Behavioral Sciences. 2nd ed Hillsdale, NJ: L. Erlbaum Associates (1988). xxi, 567 p.

[50] Taylor R Interpretation of the correlation-coefficient – a basic review. J Diagn Med Sonog (1990) 6(1):35–9. 10.1177/875647939000600106

[51] Pearson K On the criterion that a given system of deviations from the probable in the case of a correlated system of variables is such that it can be reasonably supposed to have arisen from random sampling. Philos Mag (1900) 50(302):157–75. 10.1080/14786440009463897

[52] Benjamini Y Hochberg Y Controlling the false discovery rate: a practical and powerful approach to multiple testing. J R Stat Soc Series B Stat Methodol (1995) 57(1):289–300. 10.2307/2346101

[53] Reid G Kirschner MB van Zandwijk N. Circulating microRNAs: association with disease and potential use as biomarkers. Crit Rev Oncol Hematol (2011) 80(2):193–208. 10.1016/j.critrevonc.2010.11.004 21145252

[54] Van Roosbroeck K Pollet J Calin GA. miRNAs and long noncoding RNAs as biomarkers in human diseases. Expert Rev Mol Diagn (2013) 13(2):183–204. 10.1586/erm.12.134 23477558

[55] Correia CN Nalpas NC McLoughlin KE Browne JA Gordon SV MacHugh DE Circulating microRNAs as potential biomarkers of infectious disease. Front Immunol (2017) 8:118. 10.3389/fimmu.2017.00118 28261201 PMC5311051

[56] Brochner AC Toft P. Pathophysiology of the systemic inflammatory response after major accidental trauma. Scand J Trauma Resusc Emerg Med (2009) 17:43. 10.1186/1757-7241-17-43 19754938 PMC2757019

[57] Thijs LG Hack CE Time course of cytokine levels in sepsis. Intensive Care Med (1995) 21(Suppl 2):S258–63. 10.1007/BF01740764 8636533

[58] Marques-Rocha JL Samblas M Milagro FI Bressan J Martinez JA Marti A. Noncoding RNAs, cytokines, and inflammation-related diseases. FASEB J (2015) 29(9):3595–611. 10.1096/fj.14-260323 26065857

[59] Zeng Z Gong H Li Y Jie K Ding C Shao Q Upregulation of miR-146a contributes to the suppression of inflammatory responses in LPS-induced acute lung injury. Exp Lung Res (2013) 39(7):275–82. 10.3109/01902148.2013.808285 23848342

[60] Xiao C Calado DP Galler G Thai TH Patterson HC Wang J MiR-150 controls B cell differentiation by targeting the transcription factor c-Myb. Cell (2007) 131(1):146–59. 10.1016/j.cell.2007.07.021 17923094

[61] Benz F Roy S Trautwein C Roderburg C Luedde T Circulating microRNAs as biomarkers for sepsis. Int J Mol Sci (2016) 17(1):E78 10.3390/ijms17010078 26761003 PMC4730322

[62] Xu T Zhou Q Che L Das S Wang L Jiang J Circulating miR-21, miR-378, and miR-940 increase in response to an acute exhaustive exercise in chronic heart failure patients. Oncotarget (2016) 7(11):12414–25. 10.18632/oncotarget.6966 26799589 PMC4914295

[63] Jiang X Xue M Fu Z Ji C Guo X Zhu L Insight into the effects of adipose tissue inflammation factors on miR-378 expression and the underlying mechanism. Cell Physiol Biochem (2014) 33(6):1778–88. 10.1159/000362957 24923530

[64] Gooch JL King C Francis CE Garcia PS Bai Y. Cyclosporine A alters expression of renal microRNAs: new insights into calcineurin inhibitor nephrotoxicity. PLoS One (2017) 12(4):e0175242. 10.1371/journal.pone.0175242 28414804 PMC5393575

[65] Momen-Heravi F Saha B Kodys K Catalano D Satishchandran A Szabo G. Increased number of circulating exosomes and their microRNA cargos are potential novel biomarkers in alcoholic hepatitis. J Transl Med (2015) 13:261. 10.1186/s12967-015-0623-9 26264599 PMC4533956

[66] Silakit R Loilome W Yongvanit P Thongchot S Sithithaworn P Boonmars T Urinary microRNA-192 and microRNA-21 as potential indicators for liver fluke-associated cholangiocarcinoma risk group. Parasitol Int (2017) 66(4):479–85. 10.1016/j.parint.2015.10.001 26456596

[67] Wu F Zikusoka M Trindade A Dassopoulos T Harris ML Bayless TM MicroRNAs are differentially expressed in ulcerative colitis and alter expression of macrophage inflammatory peptide-2 alpha. Gastroenterology (2008) 135(5):1624–35.e24. 10.1053/j.gastro.2008.07.068 18835392

[68] Ruckerl D Jenkins SJ Laqtom NN Gallagher IJ Sutherland TE Duncan S Induction of IL-4Ralpha-dependent microRNAs identifies PI3K/Akt signaling as essential for IL-4-driven murine macrophage proliferation in vivo. Blood (2012) 120(11):2307–16. 10.1182/blood-2012-02-408252 22855601 PMC3501641

[69] Kumarswamy R Volkmann I Thum T. Regulation and function of miRNA-21 in health and disease. RNA Biol (2011) 8(5):706–13. 10.4161/rna.8.5.16154 21712654 PMC3256347

[70] Gao Y He Y Ding J Wu K Hu B Liu Y An insertion/deletion polymorphism at miRNA-122-binding site in the interleukin-1alpha 3’ untranslated region confers risk for hepatocellular carcinoma. Carcinogenesis (2009) 30(12):2064–9. 10.1093/carcin/bgp283 19917630

[71] Chu Q Xu T miR-192 targeting IL-1RI regulates the immune response in miiuy croaker after pathogen infection in vitro and in vivo. Fish Shellfish Immunol (2016) 54:537–43. 10.1016/j.fsi.2016.05.007 27164215

[72] Wan Q Zhou Z Ding S He J. The miR-30a negatively regulates IL-17-mediated signal transduction by targeting Traf3ip2. J Interferon Cytokine Res (2015) 35(11):917–23. 10.1089/jir.2014.0146 26376209

[73] Sun Y Pan J Mao S Jin J. IL-17/miR-192/IL-17Rs regulatory feedback loop facilitates multiple myeloma progression. PLoS One (2014) 9(12):e114647. 10.1371/journal.pone.0114647 25489847 PMC4260882

[74] Vasilescu C Dragomir M Tanase M Giza D Purnichescu-Purtan R Chen M Circulating miRNAs in sepsis-A network under attack: an in-silico prediction of the potential existence of miRNA sponges in sepsis. PLoS One (2017) 12(8):e0183334. 10.1371/journal.pone.0183334 28820886 PMC5562310

[75] Xu Z Ji J Xu J Li D Shi G Liu F MiR-30a increases MDSC differentiation and immunosuppressive function by targeting SOCS3 in mice with B-cell lymphoma. FEBS J (2017) 284(15):2410–24. 10.1111/febs.14133 28605567

[76] Liu W Cao H Ye C Chang C Lu M Jing Y Hepatic miR-378 targets p110alpha and controls glucose and lipid homeostasis by modulating hepatic insulin signalling. Nat Commun (2014) 5:5684 10.1038/ncomms6684 25471065

[77] Okkenhaug K. Signaling by the phosphoinositide 3-kinase family in immune cells. Annu Rev Immunol (2013) 31:675–704. 10.1146/annurev-immunol-032712-095946 23330955 PMC4516760

[78] Wang X Wang K Han L Zhang A Shi Z Zhang K PRDM1 is directly targeted by miR-30a-5p and modulates the Wnt/beta-catenin pathway in a Dkk1-dependent manner during glioma growth. Cancer Lett (2013) 331(2):211–9. 10.1016/j.canlet.2013.01.005 23348703

[79] Wu J Zheng C Wang X Yun S Zhao Y Liu L MicroRNA-30 family members regulate calcium/calcineurin signaling in podocytes. J Clin Invest (2015) 125(11):4091–106. 10.1172/JCI81061 26436650 PMC4639992

[80] Pichiorri F Suh SS Rocci A De Luca L Taccioli C Santhanam R Downregulation of p53-inducible microRNAs 192, 194, and 215 impairs the p53/MDM2 autoregulatory loop in multiple myeloma development. Cancer Cell (2010) 18(4):367–81. 10.1016/j.ccr.2010.09.005 20951946 PMC3561766

[81] Ke S Li RC Lu J Meng FK Feng YK Fang MH. MicroRNA-192 regulates cell proliferation and cell cycle transition in acute myeloid leukemia via interaction with CCNT2. Int J Hematol (2017) 106(2):258–65. 10.1007/s12185-017-2232-2 28409330

[82] Fairbanks VF Ziesmer SC O’Brien PC. Methods for measuring plasma hemoglobin in micromolar concentration compared. Clin Chem (1992) 38(1):132–40. 1733585

[83] Noe DA Weedn V Bell WR. Direct spectrophotometry of serum hemoglobin: an Allen correction compared with a three-wavelength polychromatic analysis. Clin Chem (1984) 30(5):627–30. 6713623

[84] Gorrini C Harris IS Mak TW. Modulation of oxidative stress as an anticancer strategy. Nat Rev Drug Discov (2013) 12(12):931–47. 10.1038/nrd4002 24287781

[85] Neumann CA Krause DS Carman CV Das S Dubey DP Abraham JL Essential role for the peroxiredoxin Prdx1 in erythrocyte antioxidant defence and tumour suppression. Nature (2003) 424(6948):561–5. 10.1038/nature01819 12891360

[86] Knoops B Argyropoulou V Becker S Ferte L Kuznetsova O. Multiple roles of peroxiredoxins in inflammation. Mol Cells (2016) 39(1):60–4. 10.14348/molcells.2016.2341 26813661 PMC4749876

[87] Bandiera S Pfeffer S Baumert TF Zeisel MB miR-122 – a key factor and therapeutic target in liver disease. J Hepatol (2015) 62(2):448–57. 10.1016/j.jhep.2014.10.004 25308172

[88] Okoye IS Coomes SM Pelly VS Czieso S Papayannopoulos V Tolmachova T MicroRNA-containing T-regulatory-cell-derived exosomes suppress pathogenic T helper 1 cells. Immunity (2014) 41(1):89–103. 10.1016/j.immuni.2014.05.019 25035954 PMC4104030

