# Supplementary material for: Severity of Systemic Inflammatory Response Syndrome Affects the Blood Levels of Circulating Inflammatory-Relevant MicroRNAs
Source: Front Immunol. 2018 Feb 5;8:1977. doi: 10.3389/fimmu.2017.01977 (PMC5807656; doi:10.3389/fimmu.2017.01977)
Supplement: Supplementary file 1 [file Data_Sheet_1.PDF]

Severity of systemic inflammatory response syndrome (SIRS) affects the blood levels of circulating inflammatory-relevant miRNAs

Stefano Caserta*[1, 3], Manuela Mengozzi[1], Florian Kern[1, 2], Sarah F Newbury[1], Pietro Ghezzi[1], and Martin J Llewelyn[1, 2]

[1]Brighton and Sussex Medical School, The University of Sussex, Falmer, East Sussex, United Kingdom, BN1 9PS;
[2]Brighton and Sussex University Hospitals NHS Trust, Eastern Road, Brighton, United Kingdom BN2 5BE;
[3]Current address: School of Life Sciences, Hardy Building, The University of Hull, Hull, United Kingdom, HU6 7RX.

***Correspondence to:**
Dr Stefano Caserta. School of Life Sciences, Hardy Building, The University of Hull, Hull, United Kingdom, HU6 7RX; UK Phone: +44-(0)1482-465692; E-mail: S.Caserta@hull.ac.uk

Supplementary Material Contents:

1. **Supplementary Figure 1** **page 2**
2. **Supplementary Figure 2** **page 3**
3. **Supplementary Figure 3** **page 4**
4. **Supplementary Figure 4** **page 5**
5. **Supplementary Figure 5** **page 6**
6. **Supplementary Figure 6** **page 7**
7. **Supplementary Figure 7** **page 8**
8. **Supplementary Figure 8** **page 9**
9. **Supplementary Figure Legends** **page 10**
10. **Supplementary Table S1** **page 12**
11. **Supplementary Table S2** **page 13**
12. **Supplementary Table S3** **page 14**
13. **Supplementary Table S4** **page 15**
14. **Supplementary Table S5** **page 16**
15. **Supplementary Table S6** **page 17**
16. **Supplementary Table S7** **page 18**
17. **Supplementary Table S8** **page 20**
18. **Supplementary Materials and Methods** **page 20**
19. **Supplementary Material References** **page 22**

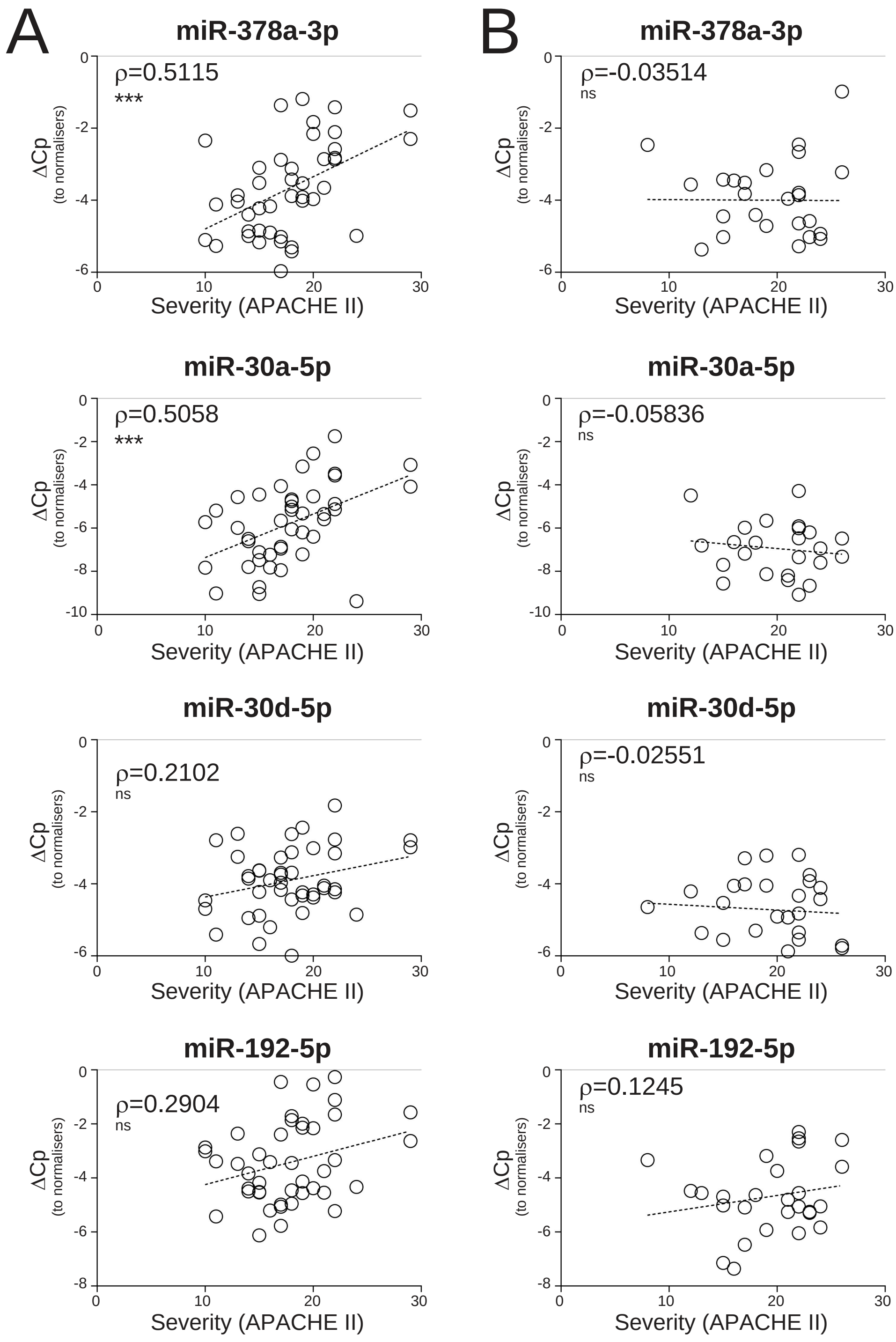

Supplementary Figure 1

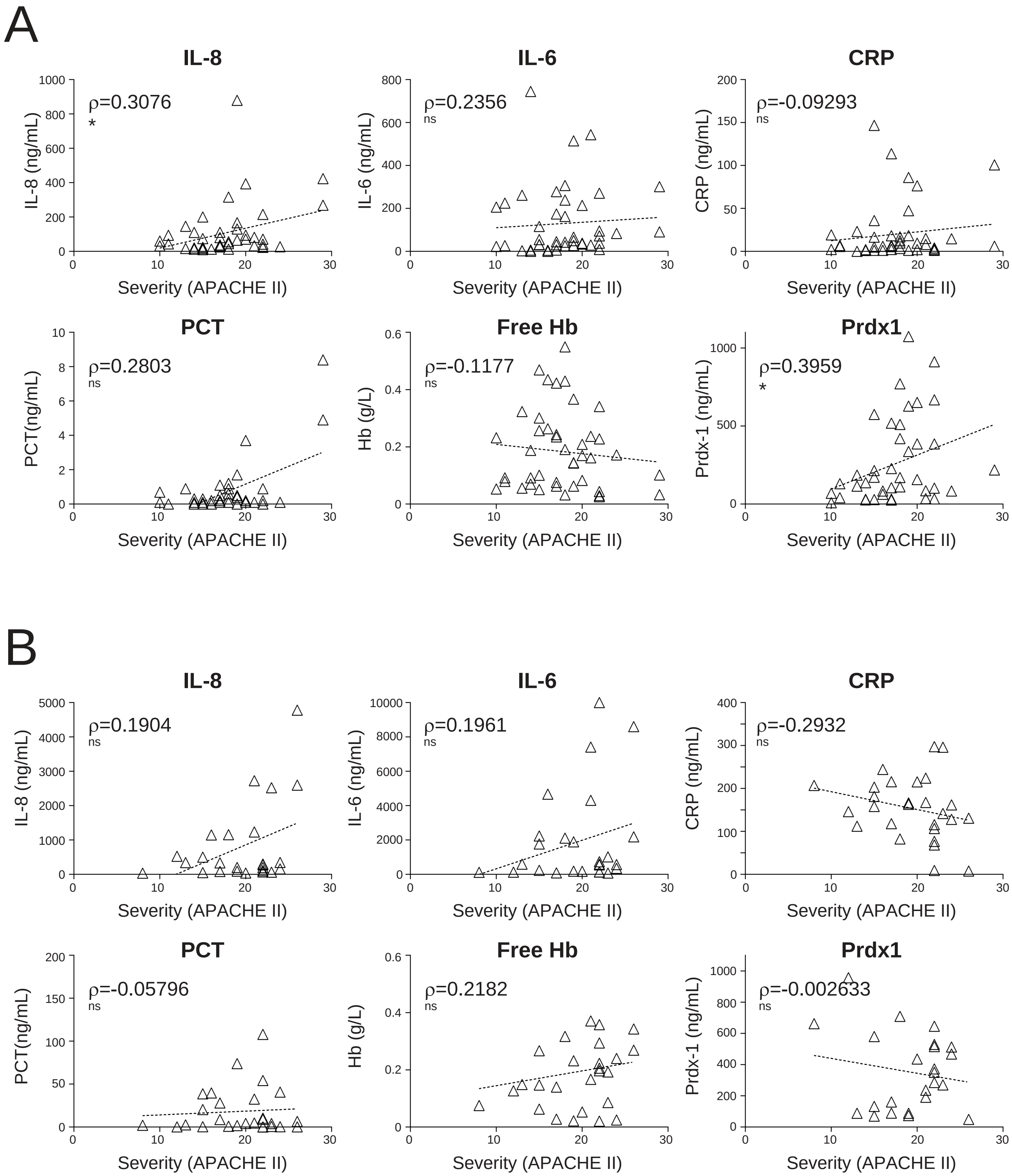

Supplementary Figure 2

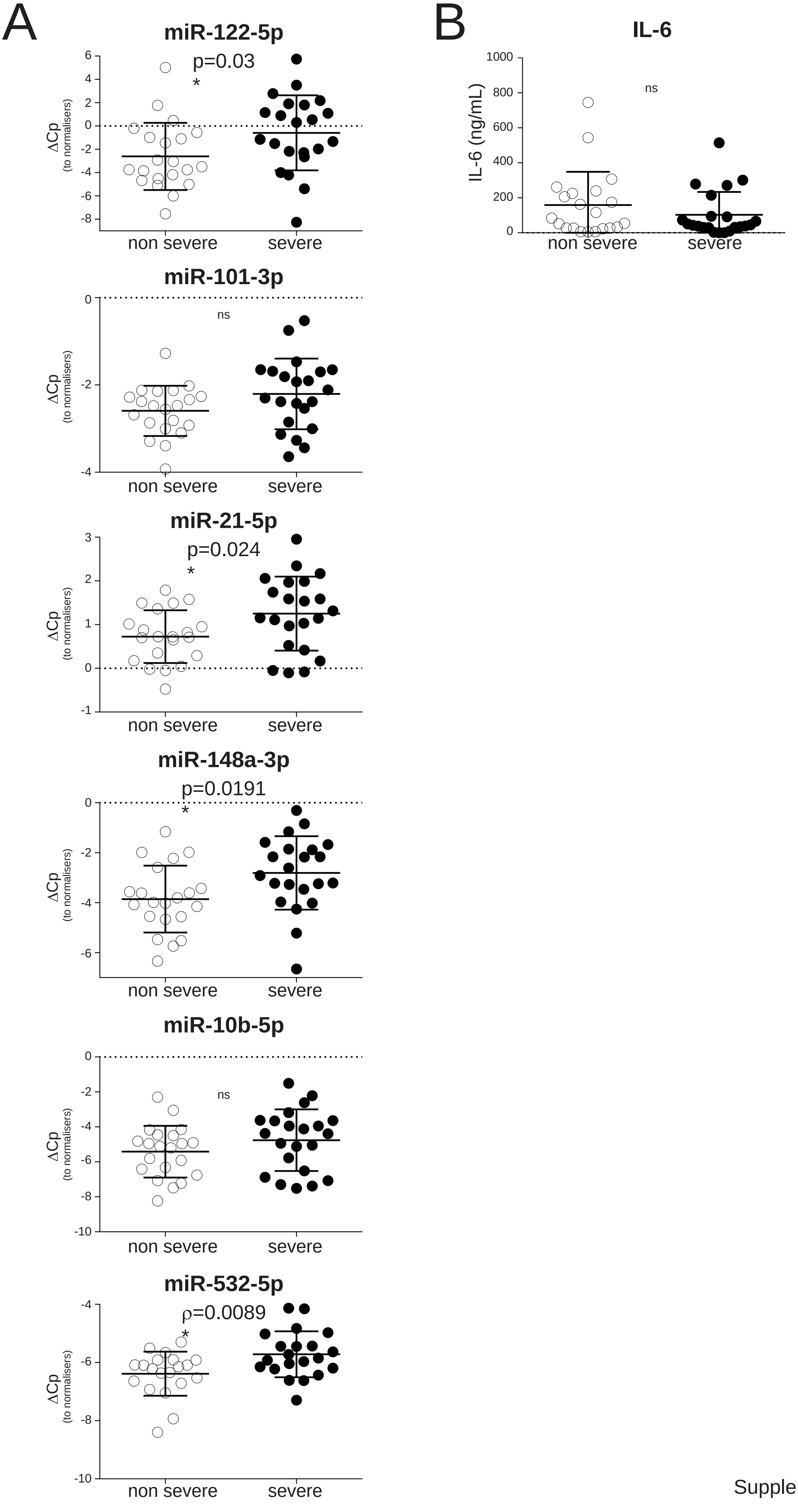

Supplementary Figure 3

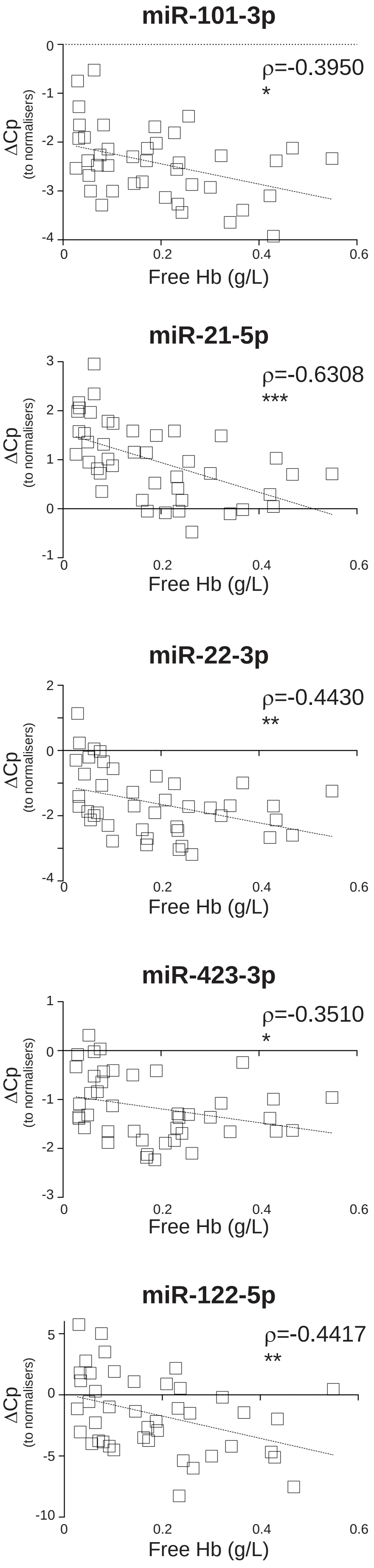

Supplementary Figure 4

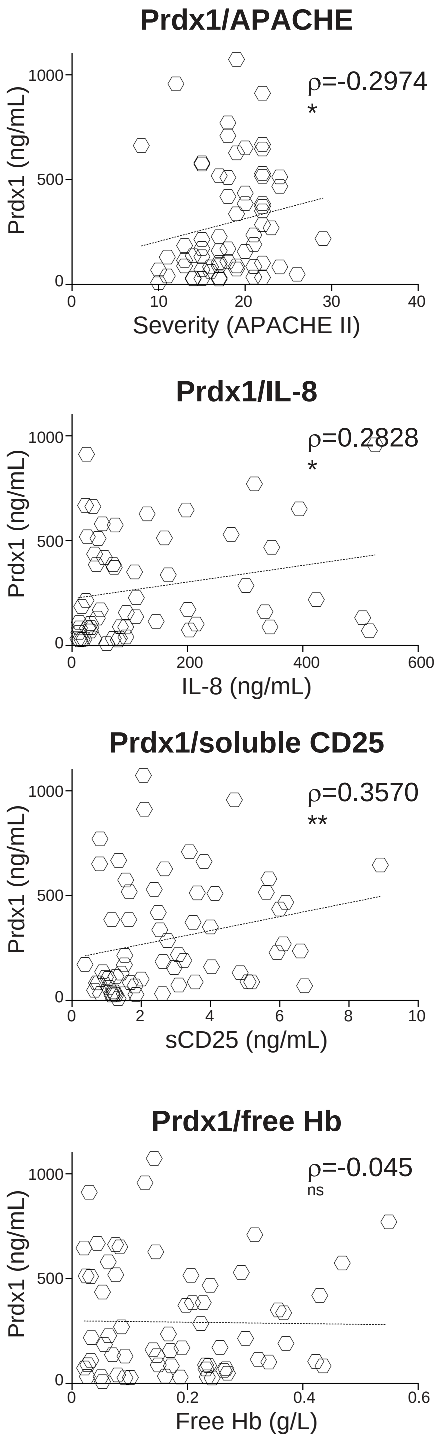

Supplementary Figure 5

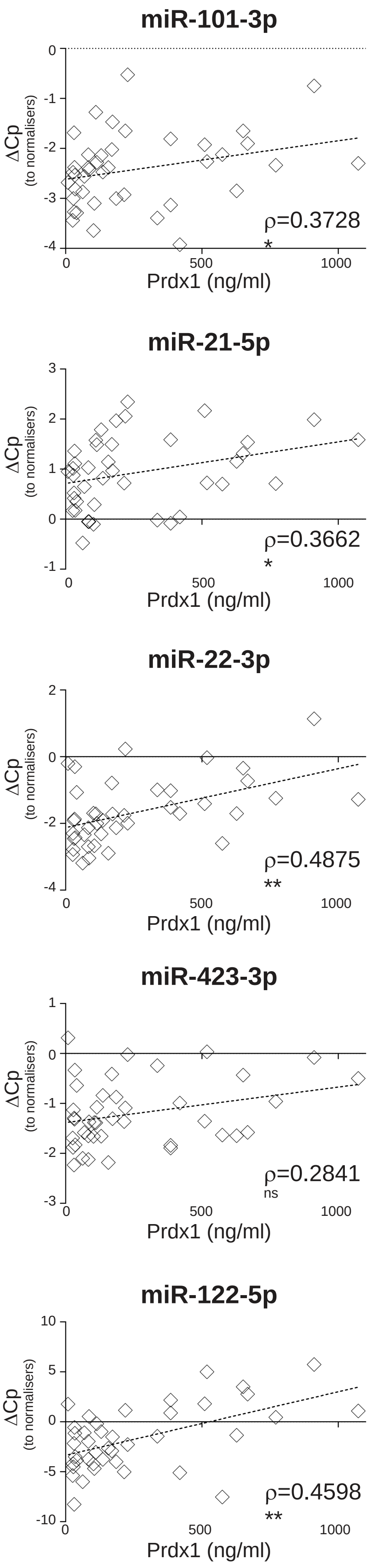

Supplementary Figure 6

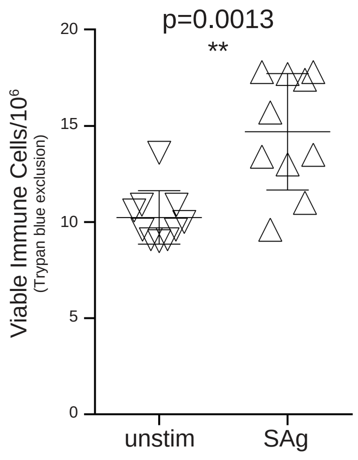

Supplementary Figure 7

miR-378a-3p

Hierarchical multiple regression levels	Variables considered	R square (% variance of dependent variable)	p (level of significance)
**1st**	Steroids; NSAIDs; Immuno-suppressants	0.127 (12.7%)	0.147 (ns)
**2nd**	SOFA (severity)	0.377(37.7%)	0.001 (***)

miR-30a-5p

Hierarchical multiple regression levels	Variables considered	R square (% variance of dependent variable)	p (level of significance)
**1st**	Steroids; NSAIDs; Immuno-suppressants	0.147 (14.7%)	0.098 (ns)
**2nd**	SOFA (severity)	0.367 (36.7%)	0.001 (***)

miR-30d-5p

Hierarchical multiple regression levels	Variables considered	R square (% variance of dependent variable)	p (level of significance)
**1st**	Steroids; NSAIDs; Immuno-suppressants	0.056 (5.6%)	0.517 (ns)
**2nd**	SOFA (severity)	0.225 (22.5%)	0.042 (*)

miR-192-5p

Hierarchical multiple regression levels	Variables considered	R square (% variance of dependent variable)	p (level of significance)
**1st**	Steroids; NSAIDs; Immuno-suppressants	0.094 (9.4%)	0.375 (ns)
**2nd**	SOFA (severity)	0.200 (20.0%)	0.070(ns)

Supplementary Figure 8

Supplementary Figure Legends

Supplementary Figure 1. CIR-miRNA levels correlate with the severity of non-infective SIRS as detected by the APACHE II score. In miRNA qPCR arrays, within each patient's specimen, Cp of a single miRNA is compared to the mean Cp of 2 normalizers (miR-486-5p and miR-320a) to give delta-Cp (dCp). dCp of non-infective SIRS **(A)** and sepsis **(B)** patients were analyzed in correlation analyses with disease severity, as determined by acute physiology and chronic health evaluation II (APACHE II) score as an alternative to the SOFA score used in Figure 1. **(A)** Non-parametric correlation of APACHE II scores with the plasma levels of miR-378a-3p, miR-30a-5p, miR-30d-5p, and miR-192-5p in non-infective SIRS patients. **(B)** Non-parametric correlation of APACHE II scores with the plasma levels of miR-378a-3p, miR-30a-5p, miR-30d-5p, and miR-192-5p in infective SIRS (sepsis) patients. Each symbol represents an individual patient. Correlation trends are shown with the linear regression model including Spearman rho (ρ) and the significances of the correlations (*, $p \leq 0.05$; **, $p \leq 0.005$; and ***, $p \leq 0.0005$ or ns, non-significant).

Supplementary Figure 2. Correlations of plasma levels of inflammatory cytokines and stress mediators with the severity of non-infective SIRS and sepsis as detected by the APACHE II score. The levels of inflammatory cytokines (interleukin-IL-8 and IL-6) and stress mediators: C-reactive protein (CRP), pro-calcitonin (PCT), free hemoglobin (Hb) and peroxiredoxin-1 (Prdx-1) were measured by ELISA in the plasma of non-infective SIRS **(A)** and sepsis **(B)** patients. Thereafter, the correlation with the severity of disease, as detected by acute physiology and chronic health evaluation II (APACHE II) score (as an alternative to the SOFA score used in Figure 1) was investigated. **(A)** Non-parametric correlation of APACHE II scores with the plasma levels of IL-8, IL-6, CRP, PCT, free Hb and Prdx-1 in non-infective SIRS patients. **(B)** Non-parametric correlation of APACHE II scores with the plasma levels of IL-8, IL-6, CRP, PCT, free Hb and Prdx-1 in sepsis patients. Each triangle represents an individual patient. Correlation trends are shown with the linear regression model including Spearman rho (ρ) and the significances of the correlations (*, $p \leq 0.05$; **, $p \leq 0.005$; and ***, $p \leq 0.0005$ or ns, non-significant).

Supplementary Figure 3. Unlike IL-6, other CIR-miRNA biomarkers discriminate the severity of non-infective SIRS. In miRNA qPCR arrays, within each patient's specimen, Cp of individual miRNAs were normalized as in Figure 1 and analyzed in patients with non-severe (open circles) and severe (filled circles) non-infective SIRS. Each symbol represents an individual patient. **(A)** Dot plots show dCp values for miR-122-5p, miR-101-3p, miR-21-5p, miR-148a-3p, miR-10b-5p and miR-532-5p in non-severe (n=21) and severe (n=22) non-infective SIRS patients, together with the level of significance. Beyond miR-378a-3p, miR-30a-5p, miR-30d-5p, and miR-192-5p (Figure 3), also miR-122-5p, miR-21-5p, miR-148a-3p and miR-532-5p significantly discriminate the severity of non-infective SIRS. **(B)** Dot plots show concentration of IL-6 in non-severe (n=21) and severe (n=22) non-infectious SIRS patients, together with the level of significance.

Supplementary Figure 4. The plasma levels of many other CIR-miRNAs inversely correlate with free Hb levels. In miRNA qPCR arrays, within each patient's specimen, Cp of individual miRNAs (open squares) were normalized as in Figure 1 and analyzed in correlation with levels of free Hb, which is derived from the lysis of red blood cells (RBCs). Each square represents an individual patient. Correlation trends are shown with the linear regression model including Spearman rho (ρ) and the significances of the correlations (*, $p \leq 0.05$; **, $p \leq 0.005$; and ***, $p \leq 0.0005$ or ns, non-significant). Beyond miR-378a-3p, miR-30a-5p, miR-30d-5p, and miR-192-5p (Figure 4), also miR-101-3p, miR-21-5p, miR-22-3p, miR-423-3p and miR-122-5p, like many others shown in Table 3, significantly correlate with free Hb.

Supplementary Figure 5. Correlations of plasma levels of Prdx-1 with IL-8, soluble CD25 and free Hb in Sepsis/SIRS. The levels of: the stress mediator, Prdx1; the inflammatory cytokine, IL-8; the soluble decoy receptor, sCD25; and free hemoglobin (Hb) were measured by ELISA in the plasma of infective and non-infective SIRS. Thereafter, the correlation of levels of Prdx-1 with IL-8, sCD25, and free Hb in parallel to the severity of disease, as detected by acute physiology and chronic health evaluation II (APACHE II) score was investigated. From the top to the bottom, graphs show the non-parametric correlation of the plasma levels of Prdx-1 with: APACHE II scores, IL-8, sCD25, and free Hb within the entire SIRS cohort. Each symbol represents an individual patient. Correlation trends are shown with the linear regression model including Spearman rho (ρ) and the significances of the correlations (*, $p \leq 0.05$; **, $p \leq 0.005$; and ***, $p \leq 0.0005$ or ns, non-significant). Prdx-1 levels tended to correlate with disease severity and levels of IL-8 and sCD25, but not free Hb.

Supplementary Figure 6. The plasma levels of many other CIR-miRNAs positively correlate with Prdx-1 levels. In miRNA qPCR arrays, within each patient's specimen, Cp of individual miRNAs (open rhombi) were normalized as specified in Figure 1 and analyzed in correlation with levels of plasma inflammatory stress marker, Prdx-1. Each symbol represents an individual patient. Correlation trends are shown with the linear regression model including Spearman rho (ρ) and the significances of the correlations (*, $p \leq 0.05$; **, $p \leq 0.005$; and ***, $p \leq 0.0005$ or ns, non-significant). Beyond miR-378a-3p, miR-30a-5p and miR-192-5p (Figure 5), many other miRNAs including miR-101-3p, miR-21-5p, miR-22-3p, and miR-122-5p significantly correlated with levels of Prdx-1 (refer to Table 4 for full list).

Supplementary Figure 7. Assessment of cell viability in cultures of PBMCs producing CIR-miRNAs. PBMCs derived from 10 individuals were freshly purified from blood and equal cells numbers were then cultured in complete media in the presence of exosome-free bovine serum, at the concentration of 2×10^6 cells/ml, in replicate wells. Half of the cultures were stimulated with the SPE-KL bacterial superantigen (SAg) from 5 days, in comparison to unstimulated controls (unstim). On day 5 cells were harvested and counted with the Trypan Blue exclusion dye. Approximately the same numbers of cells were recovered in unstimulated cultures across 10 independent biological repeats as also seen in the case of SAg activated cultures (each triangle represents a culture derived from individual donors). However, in SAg activated cultures there was approximately 1.5-fold cell-expansion.

Supplementary Figure 8. Significant effect of severity on levels of CIR-miRNAs during non-infective SIRS, after correction for medication. Hierarchical multiple regression models of the blood levels of miR-378a-3p, miR-30a-3p, miR-30d-5p and miR-192-5p were generated based on SIRS severity (SOFA), taking into account whether patients were under anti-inflammatory medication (steroids, NSAIDs and other immunosuppressants), at the time of admission (only 21% patients were taking such drugs, Table S8). In this relatively small cohort (n=43), medication dichotomous variables behaved essentially as confounding variables after they were introduced at the first level (block 1, 1st) of the regression. Thereafter, in the 2nd level (block 2, 2nd) of the regression, disease severity (SOFA) was assessed for the capacity to significantly affect the levels of blood miRNAs in SIRS. For each miRNA, tables list the variables introduced at each step (1st and 2nd) of the regression together with the resulting R square values (the percentages of the variance affected) with levels of significance after the first and the second regression steps (i.e., the total model significance). R squares values show that, in any case, medication (introduced at step 1) did not affect significantly blood levels of miRNAs ($p>0.05$, 1st). At the 2nd step, blood levels of miR-378a-3p, miR-30a-3p, and miR-30d-5p were significantly affected by severity of SIRS, accounting for 37%-20% of the variance. This suggests that severity of disease is a major factor driving the increase of CIR-miRNAs, irrespective of anti-inflammatory drug medication.

Table S1. Correlations of CIR-miRNAs with SIRS severity, as detected by APACHE II. Significant correlations with disease severity detected using the SOFA score are highlighted in bold black (SIRS) or red (sepsis) for comparison.

Micro-RNA Species[§]	APACHE II Spearman (ρ)*	Correlation significance p[#]	Benjamini Hochberg (BH) rank	BH critical value (15%)
**miR-378a-3p**	0.51145	4.56E-04	1	0.00349
**miR-30a-5p**	0.505765	0.000541	2	0.00698
**miR-122-5p**	0.365376	0.015984	3	0.01047
**miR-22-3p**	0.352489	0.020434	4	0.01395
miR-106b-3p	-0.32683	0.042274	5	0.01744
miR-30c-5p	-0.29776	0.052472	6	0.02093
**miR-192-5p**	0.290406	0.058873	7	0.02442
let7i-5p	-0.26782	0.082509	8	0.02791
miR-423-3p	-0.26607	0.084603	9	0.03140
miR-143-3p	0.248183	0.108559	10	0.03488
miR-532-5p	0.251794	0.11226	11	0.03837
**miR-101-3p**	0.217103	0.161983	12	0.04186
**miR-30d-5p**	0.210175	0.176132	13	0.04535
miR-320a	0.205505	0.186155	14	0.04884
miR-486-5p	-0.2055	0.186155	15	0.05233
miR-10b-5p	0.199895	0.198725	16	0.05581
**miR-148a-3p**	0.195878	0.208087	17	0.05930
miR-744-5p	-0.1866	0.236724	18	0.06279
miR-26a-5p	-0.17147	0.27158	19	0.06628
miR-103a-3p	-0.16791	0.281814	20	0.06977
miR-451a	-0.16343	0.295013	21	0.07326
**miR-191-5p**	-0.15138	0.332536	22	0.07674
miR-181a-5p	-0.15009	0.336716	23	0.08023
let7f-5p	-0.14994	0.33721	24	0.08372
miR-130a-3p	-0.14403	0.356821	25	0.08721
miR-941	-0.16401	0.361749	26	0.09070
miR-107	-0.12811	0.412961	27	0.09419
miR-146a-5p	-0.11204	0.474428	28	0.09767
miR-127-3p	-0.1134	0.49784	29	0.10116
miR-223-3p	-0.09476	0.545575	30	0.10465
let7a-5p	-0.09294	0.55335	31	0.10814
miR-151a-3p	-0.0921	0.55693	32	0.11163
**miR-21-5p**	0.079519	0.612242	33	0.11512
let7b-5p	0.062842	0.688911	34	0.11860
miR-10a-5p	0.04732	0.76892	35	0.12209
miR-182-5p	0.043627	0.786516	36	0.12558
miR-30e-3p	-0.04067	0.800679	37	0.12907
**miR-375**	0.033198	0.849843	38	0.13256
miR-27b-3p	0.018799	0.904757	39	0.13605

miR-423-5p	-0.01296	0.93425	40	0.13953
miR-28-3p	-0.00523	0.973445	41	0.14302
miR-92b-3p	-0.00491	0.977333	42	0.14651
miR-23a-3p	0.002577	0.986913	43	0.15000

Table S2. Correlations of CIR-miRNAs with sepsis severity, as detected by APACHE II. Significant correlations with disease severity detected using the SOFA score are highlighted in bold black (SIRS) or red (sepsis) for comparison.

**Micro-RNA species§**	**APACHE II Spearman (ρ)***	**Correlation significance p#**	**Benjamini Hochberg (BH) rank**	**BH critical value (FDR 15%)**
miR-532-5p	-0.35297	0.07694	1	0.00349
**miR-101-3p**	-0.34272	0.080122	2	0.00698
miR-10a-5p	-0.40887	0.103193	3	0.01047
miR-30e-3p	-0.33633	0.108072	4	0.01395
miR-941	-0.34139	0.129881	5	0.01744
**miR-122-5p**	0.301722	0.134137	6	0.02093
miR-107	-0.29016	0.142063	7	0.02442
miR-106b-3p	-0.29653	0.159423	8	0.02791
miR-30c-5p	-0.25788	0.194052	9	0.03140
miR-182-5p	-0.32705	0.234107	10	0.03488
miR-146a-5p	-0.22776	0.253216	11	0.03837
miR-10b-5p	-0.21358	0.29481	12	0.04186
miR-92b-3p	-0.27644	0.318577	13	0.04535
miR-26a-5p	-0.19764	0.323077	14	0.04884
miR-423-5p	-0.19487	0.330032	15	0.05233
miR-27b-3p	-0.18442	0.357119	16	0.05581
miR-23a-3p	-0.1835	0.35957	17	0.05930
miR-744-5p	-0.17074	0.425044	18	0.06279
miR-181a-5p	-0.14877	0.458954	19	0.06628
**miR-148a-3p**	0.146615	0.465548	20	0.06977
hsalet7a-5p	-0.1334	0.507118	21	0.07326
**miR-192-5p**	0.124484	0.53615	22	0.07674
hsalet7f-5p	-0.11465	0.569068	23	0.08023
miR-423-3p	-0.10604	0.598592	24	0.08372
**miR-191-5p**	-0.10512	0.601794	25	0.08721
**miR-21-5p**	0.103583	0.607145	26	0.09070
**miR-375**	0.149403	0.610212	27	0.09419
miR-127-3p	-0.11457	0.630531	28	0.09767
miR-223-3p	-0.09405	0.640762	29	0.10116
miR-130a-3p	-0.09313	0.644053	30	0.10465
miR-103a-3p	-0.09067	0.652863	31	0.10814
miR-151a-3p	-0.08606	0.669503	32	0.11163
hsalet7b-5p	0.084526	0.675084	33	0.11512
miR-320a	-0.06117	0.761833	34	0.11860

miR-486-5p	0.061166	0.761833	35	0.12209
**miR-30a-5p**	-0.05836	0.786484	36	0.12558
**miR-378a-3p**	-0.03514	0.864668	37	0.12907
**miR-30d-5p**	-0.02551	0.899487	38	0.13256
miR-451a	-0.02152	0.915169	39	0.13605
miR-143-3p	0.019672	0.922418	40	0.13953
hsalet7i-5p	0.019364	0.923626	41	0.14302
miR-28-3p	-0.00206	0.992024	42	0.14651
**miR-22-3p**	0.001844	0.992716	43	0.15000

Table S3. Correlations of CIR-miRNAs with free Hb levels in sepsis. MiRNAs significantly correlating with disease severity (as by SOFA) are highlighted in bold black (SIRS) or red (sepsis).

Micro-RNA species§	Hb Spearman correlation (ρ)*	Correlation significance p#	Benjamini Hochberg (BH) rank	BH critical value (FDR 5%)
miR-106b-3p	-0.66957	3.46E-04	1	0.00116
miR-744-5p	-0.65217	0.000554	2	0.00233
miR-151a-3p	-0.57143	0.001849	3	0.00349
miR-532-5p	-0.58085	0.001862	4	0.00465
miR-92b-3p	-0.68571	0.004772	5	0.00581
miR-130a-3p	-0.51954	0.005481	6	0.00698
miR-146a-5p	-0.50672	0.006991	7	0.00814
miR-941	-0.55065	0.009687	8	0.00930
**miR-191-5p**	-0.46642	0.014191	9	0.01047
miR-10b-5p	-0.46462	0.016788	10	0.01163
miR-10a-5p	-0.55882	0.019709	11	0.01279
miR-181a-5p	-0.35287	0.071015	12	0.01395
miR-451a	0.350427	0.073129	13	0.01512
miR-103a-3p	-0.34982	0.073665	14	0.01628
miR-28-3p	-0.35453	0.075554	15	0.01744
miR-320a	-0.34249	0.080333	16	0.01860
miR-486-5p	0.342491	0.080333	17	0.01977
miR-30c-5p	-0.29548	0.134559	18	0.02093
miR-107	-0.28999	0.142305	19	0.02209
miR-27b-3p	-0.2851	0.149455	20	0.02326
**miR-21-5p**	-0.27656	0.162581	21	0.02442
let7b-5p	0.257631	0.194502	22	0.02558
miR-143-3p	-0.25458	0.200028	23	0.02674
**miR-30a-5p**	-0.26609	0.208836	24	0.02791
let7a-5p	-0.24847	0.2114	25	0.02907
**miR-30d-5p**	-0.23016	0.248116	26	0.03023
let7f-5p	-0.21673	0.277553	27	0.03140
let7i-5p	-0.21062	0.291641	28	0.03256
miR-26a-5p	-0.21062	0.291641	29	0.03372
miR-423-5p	-0.20574	0.303231	30	0.03488

miR-423-3p	-0.1978	0.322667	31	0.03605
**miR-22-3p**	-0.18681	0.350806	32	0.03721
**miR-122-5p**	0.186325	0.362103	33	0.03837
miR-23a-3p	-0.17277	0.388811	34	0.03953
**miR-378a-3p**	-0.15487	0.44999	35	0.04070
miR-30e-3p	-0.1513	0.480346	36	0.04186
**miR-101-3p**	-0.10989	0.585311	37	0.04302
**miR-375**	-0.13846	0.636885	38	0.04419
miR-182-5p	-0.13214	0.638744	39	0.04535
miR-127-3p	-0.10677	0.654142	40	0.04651
miR-223-3p	0.043956	0.827663	41	0.04767
**miR-148a-3p**	-0.03358	0.867949	42	0.04884
**miR-192-5p**	0.029915	0.88225	43	0.05000

Table S4. Full-length version of Table 1 (SIRS). Significant correlations of CIR-miRNA levels with SIRS severity as detected by SOFA are highlighted in bold black (SIRS) or red (sepsis).

Micro-RNA species§	SOFA Spearman correlation (ρ)*	Correlation significance p#	Benjamini-Hochberg (BH) Rank	BH critical value (FDR 15%)
**miR-378a-3p**	0.491	0.00084	1*	0.00349
**miR-30a-5p**	0.433	0.00370	2*	0.00698
**miR-30d-5p**	0.412	0.00609	3*	0.01047
**miR-192-5p**	0.378	0.01253	4*	0.01395
**miR-122-5p**	0.359	0.01799	5	0.01744
**miR-101-3p**	0.351	0.02092	6	0.02093
**miR-21-5p**	0.336	0.02769	7	0.02442
**miR-148a-3p**	0.309	0.04357	8	0.02791
miR-10b-5p	0.283	0.06601	9	0.03140
miR-532-5p	0.285	0.07054	10	0.03488
**miR-22-3p**	0.268	0.08248	11	0.03837
miR-143-3p	0.266	0.08468	12	0.04186
miR-23a-3p	0.255	0.09926	13	0.04535
miR-320a	0.251	0.10514	14	0.04884
miR-486-5p	-0.251	0.10514	15	0.05233
miR-106b-3p	-0.191	0.24514	16	0.05581
let7b-5p	0.178	0.25436	17	0.05930
miR-423-3p	-0.148	0.34376	18	0.06279
let7i-5p	-0.134	0.39056	19	0.06628
miR-28-3p	0.117	0.45378	20	0.06977
miR-27b-3p	0.103	0.51175	21	0.07326
miR-130a-3p	-0.091	0.56033	22	0.07674
let7a-5p	0.089	0.57090	23	0.08023
miR-451a	-0.083	0.59466	24	0.08372
miR-10a-5p	-0.080	0.61864	25	0.08721

let7f-5p	0.077	0.62539	26	0.09070
miR-941	-0.082	0.64920	27	0.09419
miR-30e-3p	0.071	0.65837	28	0.09767
miR-744-5p	-0.066	0.67700	29	0.10116
miR-181a-5p	0.063	0.68622	30	0.10465
miR-26a-5p	0.061	0.69698	31	0.10814
miR-92b-3p	-0.067	0.69714	32	0.11163
miR-182-5p	-0.060	0.70734	33	0.11512
**miR-375**	-0.054	0.75931	34	0.11860
miR-151a-3p	0.040	0.79890	35	0.12209
miR-146a-5p	0.038	0.80829	36	0.12558
miR-223-3p	0.038	0.80829	37	0.12907
miR-30c-5p	-0.037	0.81583	38	0.13256
miR-103a-3p	-0.035	0.82375	39	0.13605
**miR-191-5p**	0.025	0.87127	40	0.13953
miR-127-3p	0.026	0.87822	41	0.14302
miR-423-5p	-0.020	0.90078	42	0.14651
miR-107	0.006	0.96827	43	0.15000

Table S5. Full-length version of Table 2 (sepsis). Significant correlations of CIR-miRNA levels with sepsis severity as detected by SOFA are highlighted in bold black (SIRS) or red (sepsis).

Micro-RNA species§	SOFA Spearman correlation (ρ)*	Correlation significance p#	Benjamini Hochberg (BH) rank	BH critical value (FDR 15%)
**miR-22-3p**	0.447	0.01942	1	0.00349
**miR-191-5p**	-0.432	0.02436	2	0.00698
**miR-375**	0.590	0.02633	3	0.01047
miR-151a-3p	-0.354	0.06990	4	0.01395
miR-146a-5p	-0.333	0.08966	5	0.01744
miR-103a-3p	-0.299	0.12992	6	0.02093
**miR-378a-3p**	0.282	0.16305	7	0.02442
let7b-5p	-0.269	0.17521	8	0.02791
**miR-122-5p**	0.262	0.19539	9	0.03140
let7i-5p	-0.252	0.20556	10	0.03488
**miR-192-5p**	0.228	0.25220	11	0.03837
miR-23a-3p	-0.224	0.26149	12	0.04186
miR-92b-3p	-0.304	0.27048	13	0.04535
miR-10a-5p	-0.275	0.28483	14	0.04884
miR-28-3p	-0.192	0.34735	15	0.05233
miR-107	-0.188	0.34852	16	0.05581
miR-423-3p	-0.178	0.37562	17	0.05930
**miR-30d-5p**	-0.161	0.42252	18	0.06279
miR-182-5p	0.220	0.43072	19	0.06628
miR-941	-0.161	0.48520	20	0.06977

miR-223-3p	-0.139	0.48880	21	0.07326
miR-130a-3p	-0.135	0.50148	22	0.07674
miR-423-5p	-0.135	0.50246	23	0.08023
**miR-101-3p**	0.132	0.51035	24	0.08372
let7f-5p	-0.129	0.52030	25	0.08721
let7a-5p	-0.121	0.54659	26	0.09070
miR-26a-5p	-0.108	0.59244	27	0.09419
miR-27b-3p	-0.106	0.59989	28	0.09767
miR-143-3p	-0.102	0.61166	29	0.10116
**miR-30a-5p**	0.108	0.61703	30	0.10465
miR-106b-3p	-0.104	0.63019	31	0.10814
**miR-148a-3p**	0.089	0.65859	32	0.11163
miR-10b-5p	0.083	0.68572	33	0.11512
miR-30e-3p	-0.078	0.71743	34	0.11860
miR-181a-5p	0.070	0.72964	35	0.12209
miR-30c-5p	-0.048	0.81357	36	0.12558
miR-532-5p	-0.047	0.81781	37	0.12907
miR-127-3p	0.051	0.82948	38	0.13256
miR-451a	0.026	0.89594	39	0.13605
miR-744-5p	-0.024	0.91287	40	0.13953
**miR-21-5p**	0.007	0.97210	41	0.14302
miR-320a	-0.007	0.97331	42	0.14651
miR-486-5p	0.007	0.97331	43	0.15000

Table S6. Full-length version of Table 3: correlations of CIR-miRNAs with free Hb levels in non-infective SIRS. MiRNAs significantly correlating with disease severity (as by SOFA) are highlighted in bold black (SIRS) or red (sepsis).

Micro-RNA species[§]	Hb Spearman correlation (ρ)*	Correlation significance p[#]	Benjamini Hochberg (BH) rank	BH critical value (FDR 5%)
**miR-21-5p**	-0.6308	5.78E-06	1	0.00116
miR-10b-5p	-0.5297693	2.59E-04	2	0.00233
miR-320a	-0.5080983	0.000504483	3	0.00349
miR-486-5p	0.5080983	0.000504483	4	0.00465
**miR-375**	-0.5562014	0.000521672	5	0.00581
miR-451a	0.5024351	0.000596273	6	0.00698
miR-30e-3p	-0.4792892	0.001521716	7	0.00814
**miR-30a-5p**	-0.4631706	0.001761638	8	0.00930
miR-92b-3p	-0.4986164	0.001966918	9	0.01047
**miR-22-3p**	-0.4430098	0.002929346	10	0.01163
**miR-122-5p**	-0.4416506	0.00302822	11	0.01279
miR-28-3p	-0.4402159	0.003135754	12	0.01395
miR-146a-5p	-0.4328161	0.00374532	13	0.01512
**miR-192-5p**	-0.4219428	0.004828522	14	0.01628
**miR-101-3p**	-0.4151471	0.005636129	15	0.01744

**miR-30d-5p**	-0.4115382	0.006110867	16	0.01860
miR-27b-3p	-0.4023106	0.007485402	17	0.01977
let7f-5p	-0.3962699	0.008523216	18	0.02093
miR-151a-3p	-0.3890965	0.009914354	19	0.02209
miR-107	-0.3855476	0.01067175	20	0.02326
miR-26a-5p	-0.380262	0.01189136	21	0.02442
miR-744-5p	-0.3832098	0.01224583	22	0.02558
**miR-378a-3p**	-0.3773172	0.01262106	23	0.02674
let7a-5p	-0.3755805	0.01306903	24	0.02791
miR-23a-3p	-0.3574584	0.0186085	25	0.02907
miR-130a-3p	-0.3562502	0.01903926	26	0.03023
miR-423-5p	-0.3510401	0.02099429	27	0.03140
miR-10a-5p	-0.3413912	0.02892798	28	0.03256
miR-103a-3p	-0.3304262	0.03045712	29	0.03372
**miR-148a-3p**	-0.3062634	0.04578071	30	0.03488
miR-143-3p	-0.2963718	0.05363466	31	0.03605
miR-941	-0.3071781	0.08205511	32	0.03721
miR-106b-3p	-0.2697505	0.09676793	33	0.03837
miR-182-5p	-0.2561087	0.1060366	34	0.03953
miR-30c-5p	-0.2478952	0.1089817	35	0.04070
miR-423-3p	-0.206894	0.1831324	36	0.04186
miR-181a-5p	-0.1957942	0.2082848	37	0.04302
**miR-191-5p**	-0.1871107	0.2295738	38	0.04419
let7b-5p	-0.1788802	0.2510852	39	0.04535
miR-127-3p	-0.1535263	0.3574384	40	0.04651
miR-532-5p	-0.1378109	0.3902074	41	0.04767
let7i-5p	-0.07029863	0.6541891	42	0.04884
miR-223-3p	0.04115226	0.7933117	43	0.05000

Table S7. Full-length version of Table 4: correlations of CIR-miRNAs with Prdx-1 levels in non-infective SIRS. MiRNAs significantly correlating with disease severity (as by SOFA) are highlighted in bold black (SIRS) or red (sepsis).

**Micro-RNA species§**	**Prdx1 Spearman correlation (ρ)***	**Correlation significance p#**	**Benjamini Hochberg (BH) rank**	**BH critical value (FDR 5%)**
**miR-192-5p**	0.590909	3.02E-05	1	0.00116
**miR-30a-5p**	0.548626	1.39E-04	2	0.00233
**miR-22-3p**	0.536243	2.10E-04	3	0.00349
**miR-122-5p**	0.505436	0.000545917	4	0.00465
**miR-148a-3p**	0.485956	0.00095437	5	0.00581
**miR-378a-3p**	0.485503	0.000966472	6	0.00698
miR-532-5p	0.465157	0.002181351	7	0.00814
miR-320a	0.453186	0.002274844	8	0.00930
miR-486-5p	-0.45319	0.002274844	9	0.01047

**miR-21-5p**	0.437632	0.003337895	10	0.01163
**miR-101-3p**	0.413168	0.005892309	11	0.01279
miR-423-5p	0.340985	0.02524517	12	0.01395
**miR-30d-5p**	0.318571	0.03733837	13	0.01512
**miR-375**	0.342017	0.04432343	14	0.01628
miR-10b-5p	0.266687	0.08386032	15	0.01744
miR-106b-3p	-0.26761	0.09954696	16	0.01860
miR-451a	-0.24524	0.1129453	17	0.01977
miR-941	0.278409	0.116678	18	0.02093
miR-143-3p	0.230746	0.1365694	19	0.02209
miR-127-3p	0.216763	0.1911489	20	0.02326
miR-146a-5p	0.200997	0.1962112	21	0.02442
miR-30e-3p	0.198781	0.2127893	22	0.02558
miR-10a-5p	0.198258	0.2140192	23	0.02674
miR-28-3p	0.191634	0.2183055	24	0.02791
miR-23a-3p	0.173815	0.2649776	25	0.02907
let7f-5p	-0.16626	0.2866147	26	0.03023
miR-130a-3p	0.165358	0.2892862	27	0.03140
miR-27b-3p	0.155542	0.3192592	28	0.03256
let7i-5p	-0.1264	0.4192846	29	0.03372
let7a-5p	-0.09619	0.5394614	30	0.03488
miR-744-5p	-0.08387	0.597466	31	0.03605
miR-103a-3p	-0.07581	0.6289866	32	0.03721
miR-92b-3p	-0.07362	0.6696049	33	0.03837
let7b-5p	-0.06312	0.6875891	34	0.03953
miR-182-5p	0.055401	0.7308226	35	0.04070
**miR-191-5p**	-0.05044	0.7480572	36	0.04186
miR-30c-5p	-0.03972	0.8003754	37	0.04302
miR-107	-0.03186	0.8392622	38	0.04419
miR-223-3p	-0.02764	0.8603651	39	0.04535
miR-26a-5p	-0.02718	0.8626321	40	0.04651
miR-423-3p	-0.02235	0.8868777	41	0.04767
miR-151a-3p	0.012534	0.9364187	42	0.04884
miR-181a-5p	0.00453	0.9769984	43	0.05000

[§]Green-shadowed cells indicate miRNAs that passed the BH correction for multiple comparisons.

*Blue and violet-shadowed cells indicate miRNAs that returned positive and negative correlations ($\rho \geq 0.2$ or $\rho \leq -0.2$), respectively.

[#]Brown and red-shadowed cells indicate miRNAs that returned significant correlations ($p \leq 0.05$) and additionally passed the correction for multiple comparisons, respectively.

Table S8. Medication at the time of admission in the study cohort. The data of medication targeting inflammation (steroids, NSAIDs and other immune-suppressants) used by patients at the time of admission is shown for non-infective SIRS and Sepsis patients. The third column (significance) shows whether the distribution differed between the two groups.

	SIRS	Sepsis	Significance
**Steroids**	4/43 (9.3%)	5/29 (17%)	ns
**NSAIDs**	3/43 (7.0%)	2/29 (6.9%)	ns
**Immuno-suppressants**	6/43 (14%)	1/29 (3.4%)	ns
**Total with anti-inflammatory drug treatment at admission**	9/43 (21%)	6/29 (21%)	ns

Supplementary Materials and Methods

Patients and healthy donors.

The patient population used in this study was previously described in detail (1) (2). Briefly, patients comprised unselected adult admissions to a mixed medical / surgical intensive / high-dependency care unit (ICU/HDU) at an English acute hospital (Brighton and Sussex University Hospitals NHS Trust). Patients were categorized as having non-infective (n=44) or infective (n=29) SIRS, following standard criteria(1). For each patient, we gathered data describing demographics, reason for admission to ICU/HDU, severity of illness (by SOFA and APACHE II scores, in the first 24 hours), comorbidities, focus of infection (for sepsis patients), and routine clinical blood test results. We defined distinct levels of non-infective SIRS severity: severe (SOFA≥6) and non-severe (SOFA≤3); patients with intermediate SOFA scores of 4-5 were excluded. Only patients with abdominal sepsis were included in this study. Blood samples were collected within <6 hours from admission and time of sample collection did not affect levels of CIR-miRNAs(2). We gathered information about any medication affecting inflammation that SIRS/sepsis patients were taking at the time of admission in ICU/HDU. A minority (21%) of patients had received previous treatment with anti-inflammatory drugs, including steroids, non-steroid anti-inflammatory drugs (NSAIDs) and other immuno-suppressants (Table S8). The number of patients taking anti-inflammatory drug medication was not significantly different in the non-infective SIRS and sepsis cohorts (Table S8). Multiple regression models designed to assess the variation in blood levels of miR-378a-3p, miR-30a-5p, miR-30d-5p and miR192-5p dependent on the severity of SIRS showed no significant contribution of anti-inflammatory drug medication at the time of admission (Supplementary Fig. 8). Healthy donors were recruited at Brighton and Sussex Medical School (BSMS) in the phlebotomy facility of the University of Sussex (Falmer, Brighton, UK). Generally healthy donors (aged 22-63; 50% males) according to criteria outlined in the National Blood Service guidelines were enrolled,

excluding pregnant women and individuals with: diabetes, asthma, past or present cancer, a known infection (e.g., HIV); blood disorders (e.g., anemia), fever, any chemotherapy, antibiotics and/or any other treatment/vaccination or long-term medication.

Haemoglobin analysis in plasma samples.

RBC lysis can bias miRNA content in plasma(3, 4). The concentration of free hemoglobin ([Hb]) was independently measured in patient plasma by the Harboe spectrophotometric method(5, 6) and hemolytic samples(7) with [Hb]>0.6g/L(8) were excluded (n=2). In parallel, we scored hemolysis in qPCR miRNA arrays as miR23a/miR451a ratio and excluded one sample that scored >7(2, 9) from further analysis.

Human samples and T cell cultures.

Fresh human PBMCs were derived from heathy donor blood after isolation by centrifugation over Ficoll-Hypaque density gradient as previously described(10). Stimulated and unstimulated cultures were seeded under identical conditions (i.e., same input; same cell concentration; exactly the same volumes). First, cells were washed in sterile PBS (ThermoFisherScientific) and counted with 0.1% Trypan Blue cell-viability exclusion dye (Sigma). Thereafter, for each condition, 20×10^6 viable PBMCs were resuspended in 10 ml (2×10^6 cells/ml) of complete media: RPMI containing 100 IU/ml penicillin, 100 µg/ml streptomycin, 2 mM L-glutamine (all from ThermoFisherScientific) and 10% of exosome-depleted, heat-deactivated fetal calf serum (FCS), to minimize the contamination with bovine blood-derived miRNAs in our analyses (System Biosciences). PBMCs were then seeded in 24 well-plates in replicate wells and stimulated with the bacterial superantigen (SAg), streptococcal pyrogenic exotoxin K/L (SPE-K/L, purified from *Escherichia coli* transfected with a spe-K/L-expressing vector(11), a gift from Prof Thomas Proft - University of Auckland, New Zealand) as described before(10), in parallel to unstimulated control cultures. After 5 days, cell cultures were harvested and viability of the cells was tested with Trypan-blue (0.1%) dye exclusion (Supplementary Fig. 7). Equal volumes of culture supernatants were then recovered, frozen at -80°C and total RNA was then extracted as described below.

RNA extraction and microRNA real-time qPCR array.

Total plasma and supernatant RNA was extracted using the miRCURY™ RNA isolation - biofluids kit (Exiqon, Denmark). After thawing on ice, an RNA spike-in template mixture was added to the samples. Eight ml of supernatant per sample was mixed with 2.4 ml of Lysis solution BF containing 16.67 µg/mL of MS2 bacteriophage RNA (UniSp6), prior to proceeding to the purification as by manufacturer instructions. Plasma was thawed on ice and centrifuged (3000g, 5 min, 4°C). For each sample, plasma (200 µL) was mixed with 60 µl of Lysis solution BF containing 1 µg carrier-RNA per 60µl Lysis Solution BF and RNA spike-in template mixture (UniSp4, UniSp3 and UniSp6). Samples were vortexed briefly and incubated for 3 min at room temperature, before adding 20 µL Protein Precipitation solution BF. Samples were vortexed again, incubated for 1 min at room temperature and centrifuged (11000g, 3 min). Clear supernatants were mixed with isopropanol (270 µL, SIGMA), briefly vortexed and loaded onto binding columns. After multiple washes, total RNA was eluted in RNase-free H_2O by centrifugation (11000g) and stored at -80°C.

RNA (2 µl) was reverse transcribed (RT) using the miRCURY LNA™ Universal RT microRNA PCR, Polyadenylation and cDNA synthesis kit (Exiqon). cDNA (1:50) was assayed in qPCR as by the miRCURY LNA™ Universal RT microRNA PCR, Polyadenylation and cDNA synthesis method (Exiqon). For plasma samples derived from patients, each microRNA was assayed by qPCR (microRNA Ready-to-Use PCR, Pick-&-Mix using ExiLENT SYBR® Green master mix) in 2

independent technical repeats including negative controls (no-template from the RT reaction) using a LightCycler® 480 Real-Time PCR System (Roche). In each experimental group, ≥8 biological replicates were included. Superantigen stimulation experiments were performed as 10 biological replicates. The amplification was performed in a LightCycler® 480 Real-Time PCR System (Roche) in 384-well plates. The amplification curves were analyzed using the Roche LC software, both for determination of Cq (2nd derivative method) and for melting curve analysis. Amplification efficiency was calculated using a linear regression method. All assays were inspected for distinct melting curves and the Tm was confirmed to be within known specifications for the assay. Assays returning 3 crossing point (Cp) values less than the negative control and Cp<37 were accepted. Excluding spike-ins and no template controls, we analyzed 44 CIR-miRNAs in Q-PCR arrays from sepsis/SIRS patients. Although out of these, most miRNAs (33 and 29 respectively in SIRS and sepsis patients, including normalizer miRNAs) were detectable in all individuals, some miRNAs were not expressed in all individuals (i.e., Cp>37), affecting n in correlation analyses as indicated in individual figure legends. Further, miR-103b assay did not return acceptable Cp values in any donor and was excluded, leading to final 43 CIR-miRNA species analyzed. The most significantly affected miRNAs (miR-378a-3p, miR-30a-5p, miR-30d-5p, and miR-192-5p) were expressed in (89-100%) of sepsis/SIRS individuals. The stability values of candidate normalizers were assessed using the 'NormFinder' software(12). Any qPCR data was normalized to the average Cp of internal normalizers (miR-320a and miR-485-5p (2)), detected in all plasma samples as described previously (2) or the Cp of normalizer spike-in ((UniSp6), in the case of culture supernatants (delta Cp, dCp=normalizer Cp–assay Cp) as indicated in individual experiments. Hence relative to the normalizer/s, dCp values that become more positive correspond to an increase in the abundancy of specific miRNA species.

Supplementary Material References

1. Llewelyn MJ, Berger M, Gregory M, Ramaiah R, Taylor AL, Curdt I, et al. Sepsis biomarkers in unselected patients on admission to intensive or high-dependency care. *Crit Care* (2013) 17(2):R60. Epub 2013/03/28. doi: 10.1186/cc12588. PubMed PMID: 23531337; PubMed Central PMCID: PMC3672658.
2. Caserta S, Kern F, Cohen J, Drage S, Newbury SF, Llewelyn MJ. Circulating Plasma microRNAs can differentiate Human Sepsis and Systemic Inflammatory Response Syndrome (SIRS). *Sci Rep* (2016) 6:28006. doi: 10.1038/srep28006. PubMed PMID: 27320175; PubMed Central PMCID: PMC4913253.
3. Pritchard CC, Kroh E, Wood B, Arroyo JD, Dougherty KJ, Miyaji MM, et al. Blood cell origin of circulating microRNAs: a cautionary note for cancer biomarker studies. *Cancer Prev Res (Phila)* (2012) 5(3):492-7. doi: 10.1158/1940-6207.CAPR-11-0370. PubMed PMID: 22158052; PubMed Central PMCID: PMC4186243.
4. Kirschner MB, Kao SC, Edelman JJ, Armstrong NJ, Vallely MP, van Zandwijk N, et al. Haemolysis during sample preparation alters microRNA content of plasma. *PLoS One* (2011) 6(9):e24145. doi: 10.1371/journal.pone.0024145. PubMed PMID: 21909417; PubMed Central PMCID: PMC3164711.
5. Harboe M. A method for determination of hemoglobin in plasma by near-ultraviolet spectrophotometry. *Scand J Clin Lab Invest* (1959) 11:66-70. doi: 10.3109/00365515909060410. PubMed PMID: 13646603.
6. Adamzik M, Hamburger T, Petrat F, Peters J, de Groot H, Hartmann M. Free hemoglobin concentration in severe sepsis: methods of measurement and prediction of outcome. *Crit Care* (2012) 16(4):R125. doi: 10.1186/cc11425. PubMed PMID: 22800762; PubMed Central PMCID: PMC3580706.

7. Han V, Serrano K, Devine DV. A comparative study of common techniques used to measure haemolysis in stored red cell concentrates. *Vox Sang* (2010) 98(2):116-23. doi: 10.1111/j.1423-0410.2009.01249.x. PubMed PMID: 19719459.

8. Lippi G, Salvagno GL, Montagnana M, Brocco G, Guidi GC. Influence of hemolysis on routine clinical chemistry testing. *Clin Chem Lab Med* (2006) 44(3):311-6. doi: 10.1515/CCLM.2006.054. PubMed PMID: 16519604.

9. Blondal T, Jensby Nielsen S, Baker A, Andreasen D, Mouritzen P, Wrang Teilum M, et al. Assessing sample and miRNA profile quality in serum and plasma or other biofluids. *Methods* (2013) 59(1):S1-6. doi: 10.1016/j.ymeth.2012.09.015. PubMed PMID: 23036329.

10. Caserta S, Taylor AL, Terrazzini N, Llewelyn MJ. Induction of Human Regulatory T Cells with Bacterial Superantigens. *Methods Mol Biol* (2016) 1396:181-206. doi: 10.1007/978-1-4939-3344-0_16. PubMed PMID: 26676048.

11. Taylor AL, Llewelyn MJ. Superantigen-induced proliferation of human CD4+CD25- T cells is followed by a switch to a functional regulatory phenotype. *J Immunol* (2010) 185(11):6591-8. doi: 10.4049/jimmunol.1002416. PubMed PMID: 21048104.

12. Andersen CL, Jensen JL, Orntoft TF. Normalization of real-time quantitative reverse transcription-PCR data: a model-based variance estimation approach to identify genes suited for normalization, applied to bladder and colon cancer data sets. *Cancer Res* (2004) 64(15):5245-50. doi: 10.1158/0008-5472.CAN-04-0496. PubMed PMID: 15289330.
